# The exposure risk to COVID-19 in most affected countries: A vulnerability assessment model

**DOI:** 10.1371/journal.pone.0248075

**Published:** 2021-03-04

**Authors:** Adriana Nascimento Santos Cartaxo, Francisco Iran Cartaxo Barbosa, Paulo Henrique de Souza Bermejo, Marina Figueiredo Moreira, David Nadler Prata

**Affiliations:** 1 General-Coordination of the Health Industrial Complex, Secretariat of Science Technology Innovation and Strategic Supplies in Health, Ministry of Health, Brasília, Federal District, Brazil; 2 Medical Equipment Office, Brazil Health Surveillance Agency – ANVISA, Brasília, Federal District, Brazil; 3 Department of Administration, University of Brasilia, Brasília, Federal District, Brazil; 4 Business School, University of Brasilia, Brasília, Federal District, Brazil; 5 Department of Computation Modelling, Institute of Regional Development, Federal University of Tocantins, Palmas, Tocantins, Brazil; Institute for Advanced Sustainability Studies, GERMANY

## Abstract

The world is facing the coronavirus pandemic (COVID-19), which began in China. By August 18, 2020, the United States, Brazil, and India were the most affected countries. Health infrastructure and socioeconomic vulnerabilities may be affecting the response capacities of these countries. We compared official indicators to identify which vulnerabilities better determined the exposure risk to COVID-19 in both the most and least affected countries. To achieve this purpose, we collected indicators from the Infectious Disease Vulnerability Index (IDVI), the World Health Organization (WHO), the World Bank, and the Brazilian Geography and Statistics Institute (IBGE). All indicators were normalized to facilitate comparisons. Speed, incidence, and population were used to identify the groups of countries with the highest and lowest risks of infection. Countries’ response capacities were determined based on socioeconomic, political, and health infrastructure conditions. Vulnerabilities were identified based on the indicator sensitivity. The highest-risk group included the U.S., Brazil, and India, whereas the lowest-risk group (with the largest population by continent) consisted of China, New Zealand, and Germany. The high-sensitivity cluster had 18 indicators (50% extra IDVI), such as merchandise trade, immunization, public services, maternal mortality, life expectancy at birth, hospital beds, GINI index, adolescent fertility, governance, political stability, transparency/corruption, industry, and water supply. The greatest vulnerability of the highest-risk group was related first to economic factors (merchandise trade), followed by public health (immunization), highlighting global dependence on Chinese trade, such as protective materials, equipment, and diagnostic tests. However, domestic political factors had more indicators, beginning with high sensitivity and followed by healthcare and economic conditions, which signified a lesser capacity to guide, coordinate, and supply the population with protective measures, such as social distancing.

## Introduction

The WHO had initially declared the outbreak to be a public health emergency of international concern, advising countries to implement strong measures for early detection of the disease and to promote adequate social distancing, emphasizing the urgent needs for research acceleration and vaccine development, but they declared the novel coronavirus (COVID-19) a pandemic on March 11, 2020 [1]. However, all continents currently have countries with identified community transmission, registering around 22 million cases and 775 thousand deaths as of Aug 18, 2020, with the highest of these statistics reported in the United States of America, Brazil, India, and Russia (the U.S. and Brazil have each reported more than a million cases) [2].

To effectively curb the spread of the virus, several measures have been implemented, such as increasing reserves of personal protective equipment and establishing effective communication [3–6]. In addition, the WHO has recommended app-based tracing, mass testing, and health systems equipped with ventilation equipment [7, 8].

In order to measure the efficiency of such actions, Kandel and colleagues reviewed the response capacity of 182 countries, using indicators from the State Parties Annual Reporting (SPAR) and identified that countries could widely vary in their capabilities [9, 10]. Alongside this research, Gilbert and colleagues estimated the risk of virus importation in Africa with indicators from the SPAR and added the Infectious Disease Vulnerability Index (IDVI) to explain certain vulnerabilities [3, 11].

The results of these studies have shown that COVID-19 has not solely affected countries with the lowest response capacities and greatest vulnerabilities—such as the scarcity of resources—as first imagined. However, no study predicted that rich countries with high per capita income would be as or more affected than those countries originally thought to be at higher risk.

To better understand this phenomenon, this study proposes a “different risk estimation approach” as tested by Gilbert and colleagues [3], “including vulnerabilities due to socioeconomic variables…and lack of health infrastructure…triangulated with the latest risk assessments available for COVID-19” as suggested by Kandel and colleagues [9].

This study assessed the exposure risk to COVID-19 by comparing the vulnerabilities of the group of countries that were most affected by the disease with those of the least-affected group. In order to achieve this objective, the countries were grouped by risk, determined using the speed and the incidence of cases in their populations. Vulnerabilities were identified by calculating the indicator sensitivity. The indicators were referenced internationally by the IDVI, the WHO, and the World Bank, as well as the IBGE (which aggregates several official sources with data that is sometimes more up-to-date).

## Materials and methods

### The exposure risk to COVID-19 in the population of each country

The exposure risk to COVID-19 mirrors a country’s ability to cope with the pandemic, measured by both the speed and the incidence of cases.

The data included all confirmed cases reported from December 31, 2019, to August 5, 2020, from database of the European Centre for Disease Prevention and Control (ECDC) [2]. The downloadable ECDC data file is updated daily and contains the latest available public data, with the number of new cases reported per day and per country. Every day, a team of epidemiologists screens relevant sources to collect the latest figures, based on reports from health authorities worldwide. This source provides data for all countries and territories in an easily accessible spreadsheet.

**Fig 1:**
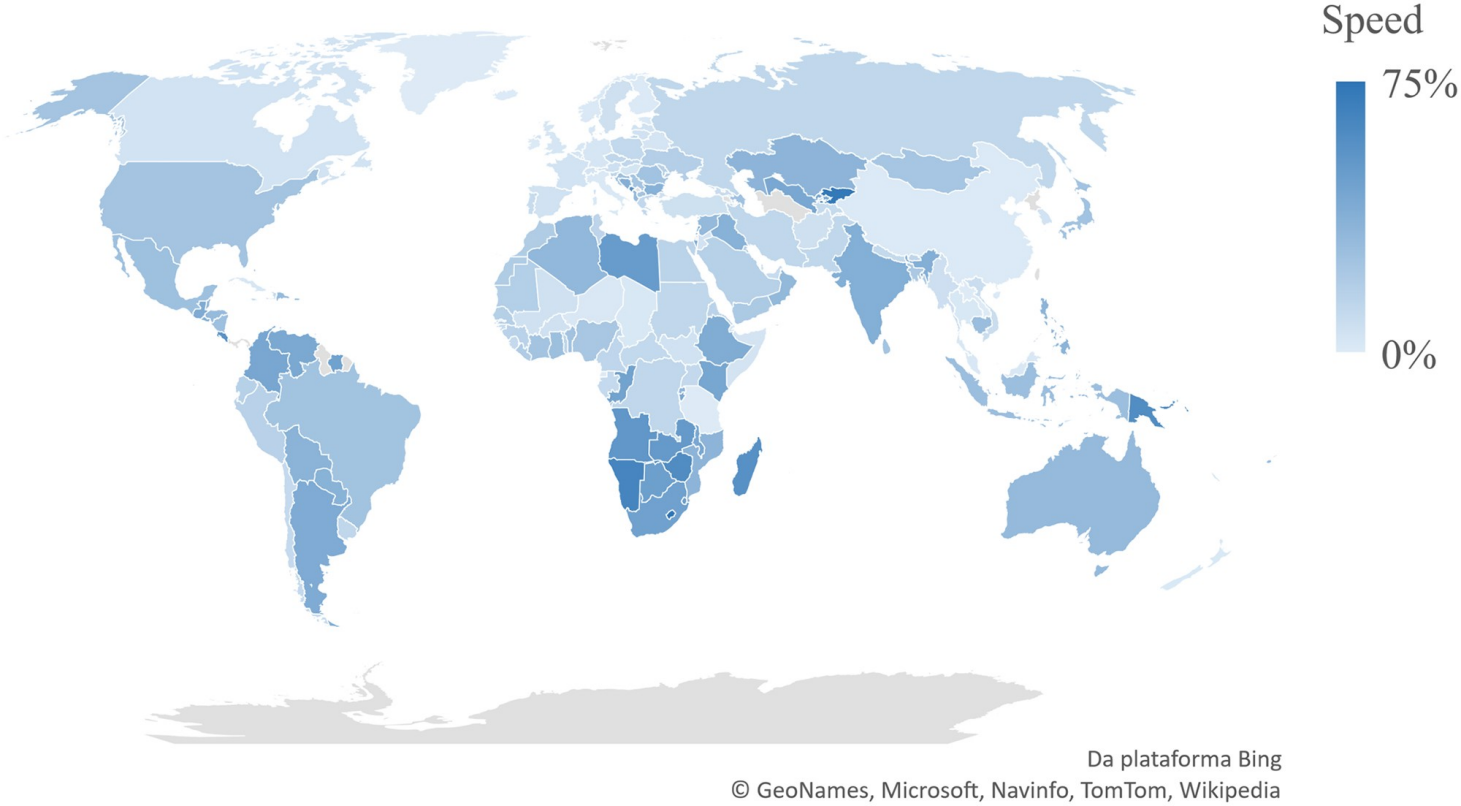
Speed of COVID-19 spreading as of August 5, 2020. Speed was calculated as incidence per country by August 5 minus incidence by July 21 divided by incidence by August 5. In grey are countries without data. Microsoft product screenshots reprinted with permission from Microsoft Corporation.

The speed was the variation in incidence per capita in the last 15 days, from July 21 to August 5, 2020. Speed and incidence were calculated based on the sum of all daily cases in each country (Fig 1). All countries’ data is found in the S1 File.

**Fig 2:**
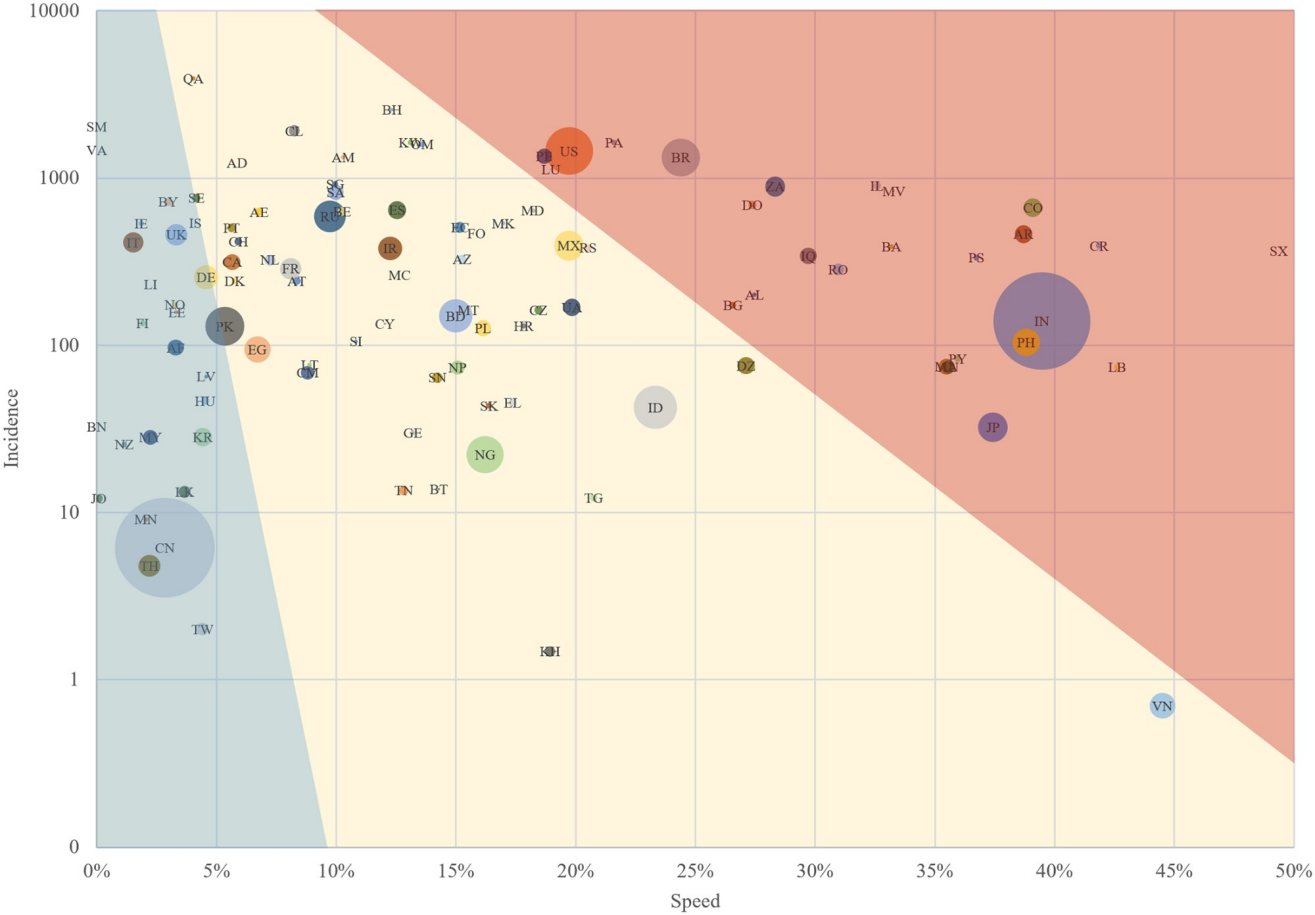
Incidence as a function of speed of COVID-19 in countries as by August 5, 2020. 106 countries with first case reported before March 11, 2020. The area of circles is proportional to country population. The red area contains the cluster of counties with the highest exposure risk to COVID-19. The blue area contains the cluster of countries with lowest risk as by August 5, 2020.

Then, we considered only countries with the first case reported before March 11, 2020, when the pandemic was declared (Fig 2), totalizing 106 countries. These countries were separated in clusters. The clusters with highest and lowest exposure risk have 26 countries each. The exposure risk was calculated using the base ten’ logarithm of the incidence per country and territories (normalized) times the speed (normalized). Exposure risk is a real factor based on the conditions of incidence (factor indicating the damage that has already occurred) and speed of cases increase (factor indicating the rate of increase in the damage that has occurred), which reflects the country’s ability to deal with the pandemic at the time considered.

Each cluster was ordered by potential damage due to exposure risk of COVID-19. The potential damage was calculated as exposure risk times population normalized. Potential damage indicates the degree of damage that a country can generate in relation to other countries, as it includes the total relative population of the country submitted to the conditions of exposure risk at the time considered.

The first country with highest potential damage was India (0.29), followed by the U.S. (0.05), Brazil (0.04), Philippines (0.02), Japan (0.02) and 21 others countries (0.07 in total). Therefore, the fourth country had half of the potential damage to the third.

The first three countries have more than 75% of the group population. Considering that, these three countries with the highest potential damage due to exposure risk to COVID-19 (i.e., the U.S., Brazil, and India) were identified according to speed of infection, incidence of infection, and population size.

This way, we also selected three countries in the cluster with lowest exposure risk. The three countries with the lowest potential damage due to exposure risk to COVID-19 were selected according to the same criteria but a country with a largest population by continent. These countries were China (0.011), New Zealand (0.00002), and Germany (0.002). They have more than 77% of the group population. The largest population criterion was selected in order to analyze the control capacities of these countries.

This quantitative study used the method adopted by Gilbert and colleagues: “Similarity between exposure profiles of different countries was quantified with entropy-based metrics [12], and used to group countries with similar…patterns via agglomerative clustering” [3]. This entropy technique was applied in this study to group similar countries and indicators.

### Indicator sensitivity as a vulnerability metric for countries

This approach led Gilbert and colleagues to introduce the IDVI index as a synthetic metric of vulnerability. But in this study, we used the indicators by IDVI separately. We organized a database based on IDVI indicators, with the addition of others from the IBGE, World Bank, and WHO databases. The new indicators were classified into the IDVI factors.

In order to build the dataset, we collected the most recent information about the indicators. Fourteen indicators were used from the IBGE database, including the following categories: social, economy, population, and health [13]. Regarding the World Bank database, fourteen indicators were also used, taken from categories such as worldview, people, environment, economy, states and markets, and global links, as well as the GINI index [14]. From the WHO Observatory database, six indicators from the health theme were used [15, 16]. Outdate data and other indicators outside the context of this study were excluded.

This new set of indicators (Box) was used to analyze the countries’ response capacities to the exposure risk to COVID-19, considering socioeconomic, political, and health infrastructure conditions.

**Box. Indicators selected.**

1 DM—Population density (IBGE)

2 DM—Population living in urban areas (IBGE)

3 DM—Average annual population growth rate (IBGE)

4 DM—Literacy rate—population aged 15 and over (IBGE)

5 DM—Gross enrollment rate all school levels (IBGE) (*)

6 DM—Population growth (annual % [WB]) (*)

7 DM—Net migration (thousands [WB])

8 DM—Population median age (years (WHO)) (*)

9 DM—Income share held by lowest 20% (WB) (*)

10 DM—Fertility rate, total (births per woman [WB]) (*)

11 DM—Adolescent fertility rate (births per 1000 women ages 15–19 [WB]) (*)

12 DM—Life expectancy at birth (IBGE) (*)

13 DM—Human Development Index (IBGE) (*)

14 EC—GDP per capita (IBGE)

15 EC—GDP per capita growth (annual % [WB])

16 EC—GDP growth (annual % [WB]) (*)

17 EC—Inflation, GDP deflator (annual % [WB]) (*)

18 EC—Poverty headcount ratio at $1.90 a day (2011 PPP (% of population [WB])

19 EC—Partner-nation transportation infrastructure: logistics performance index: overall (1 = low to 5 = high [WB])

20 EC—Partner-nation transportation infrastructure: roads paved (% of total roads [Chartsbin])

21 EC—Agriculture, forestry, and fishing, value added (% of GDP [WB]) (*)

22 EC—Industry (including construction), value added (% of GDP [WB]) (*)

23 EC—Index of Technological Sophistication (OECD)

24 EC—High-technology exports (% of manufactured exports [WB]) (*)

25 EC—Investments in research and development (IBGE) (*)

26 EC—Mobile cellular subscriptions (per 100 people [WB])

27 EC—Individuals using the Internet (% of population [WB])

28 EC—Tourist arrivals (IBGE) (*)

29 EC—Public spending on education (IBGE) (*)

30 EC—Merchandise trade (% of GDP [WB]) (*)

31 EC—GINI index (WB estimate) (*)

32 HC—Skilled health professional density (per 10 000 population)—latest available year (WHO)

33 HC—Current health expenditure (% of GDP [WB])

34 HC—Hospital beds (per 10 000 population [WHO])

35 HC—Mortality rate, infant (per 1,000 live births [WB])

36 HC—Mortality rate, under-5 (per 1,000 live births [WB]) (*)

37 HC—Maternal mortality ratio (per 100 000 live births [WHO]) (*)

38 HC—Dietary energy supply (IBGE) (*)

39 HC—Prevalence of undernourishment (IBGE) (*)

40 PH—Immunization, measles (% of children ages 12–23 months [WB])

41 PH—Water supply (IBGE)

42 PH—Sanitation (IBGE)

43 PH—Basic public health infrastructure (member of the International Association of National Public Health Institutes)

44 PH—UHC index of essential service coverage (%)—latest available year (scaled [WHO]) (*)

45 PH—Domestic General Government Health Expenditure (GGHE-D) as % GDP (WHO) (*)

46 PH—Government Health Expenditure (% GDP) (IBGE) (*)

47 PH—GHSA action packages (Country leading or contributing to >1 GHSA action package [binary; 1 = yes (good)])

48 DD—Average precipitation in depth (mm per year [WB])

49 DD—Annual average temperature (high = bad; flip measure value [Weatherbase])

50 DD—Agricultural land (% of land area [WB])

51 DD—Forest area (% of land area [WB])

52 DD—Global deforestation rates (% [Global Forest Watch])

53 DD—Energy use (kg of oil equivalent per capita [WB]) (*)

54 DD—CO2 emissions (metric tons per capita [WB]) (*)

55 PD—Worldwide Governance Indicator: Government Effectiveness (WB)

56 PD—Worldwide Governance Indicator: Regulatory Quality (WB)

57 PD—Worldwide Governance Indicator: Rule of Law Index (WB)

58 PD—Worldwide Governance Indicator: Political Stability and Absence of Violence (WB)

59 PD—Transparency International Corruption Perceptions Index

60 PD—Public Services (Fragile States Index) (*)

61 PD—State Legitimacy (Fragile States Index) (*)

62 PD—Democracy (Polity IV Project Democracy Index [Center for Systemic Peace])

63 PD—Fragility Index (Center for Systemic Peace)

64 PD—Human rights (Amnesty International Political Terror Scale)

65 PI—WB Net Official Development Assistance per capita

66 PI—WB Net Official Development Assistance received (% gross national income)

67 PI—WB Net financial flows, multilateral (current US$) (*)

*Socioeconomic*: *DM = Socio-demographic; EC = Economic*

*Health Infrastructure*: *EC = Healthcare; PH = Public Health; DD = Disease Dynamics*

*Political conditions*: *PD = Political–Domestic; PI = Political International*

(*) = *New indicators (30)*; *WB = World Bank*

The indicators were normalized (ranging from 0 [bad] to 1 [good]) and ordered (position from 1 [first = good] to 6 [sixth = bad]) to allow a comparative analysis among the six countries to specifically identify the sensitivity of each indicator. The comparative analysis aimed to identify the most significant indicators to better determine the response capacity of each country.

The indicator sensitivity (S) to the pandemic control was the sum of the relevant positions of the country groups regarding the indicator (Relevant Position of the Group to Indicator—RPGI) and the indicator relevance to discrimination between the lowest and highest risk groups (Relevance of the Indicator in the Group—RIG divided by ten). The decimal point of the indicator sensitivity allowed a fine-grained classification.

The RPGI was calculated as the difference between the sum of the positions from each country in the highest risk group (the U.S., Brazil, and India) by the sum of the positions from each country in the lowest risk group (China, New Zealand, and Germany). This result was adjusted in order to obtain positive values.

The indicator variation (ranging from 0 to 1) was used to identify their relevance. The RIG was calculated as the difference between the sum of each indicator for the countries in the lowest risk group by the sum of those in the highest risk group.

## Results

### Countries with high exposure risk to COVID-19

**Fig 3:**
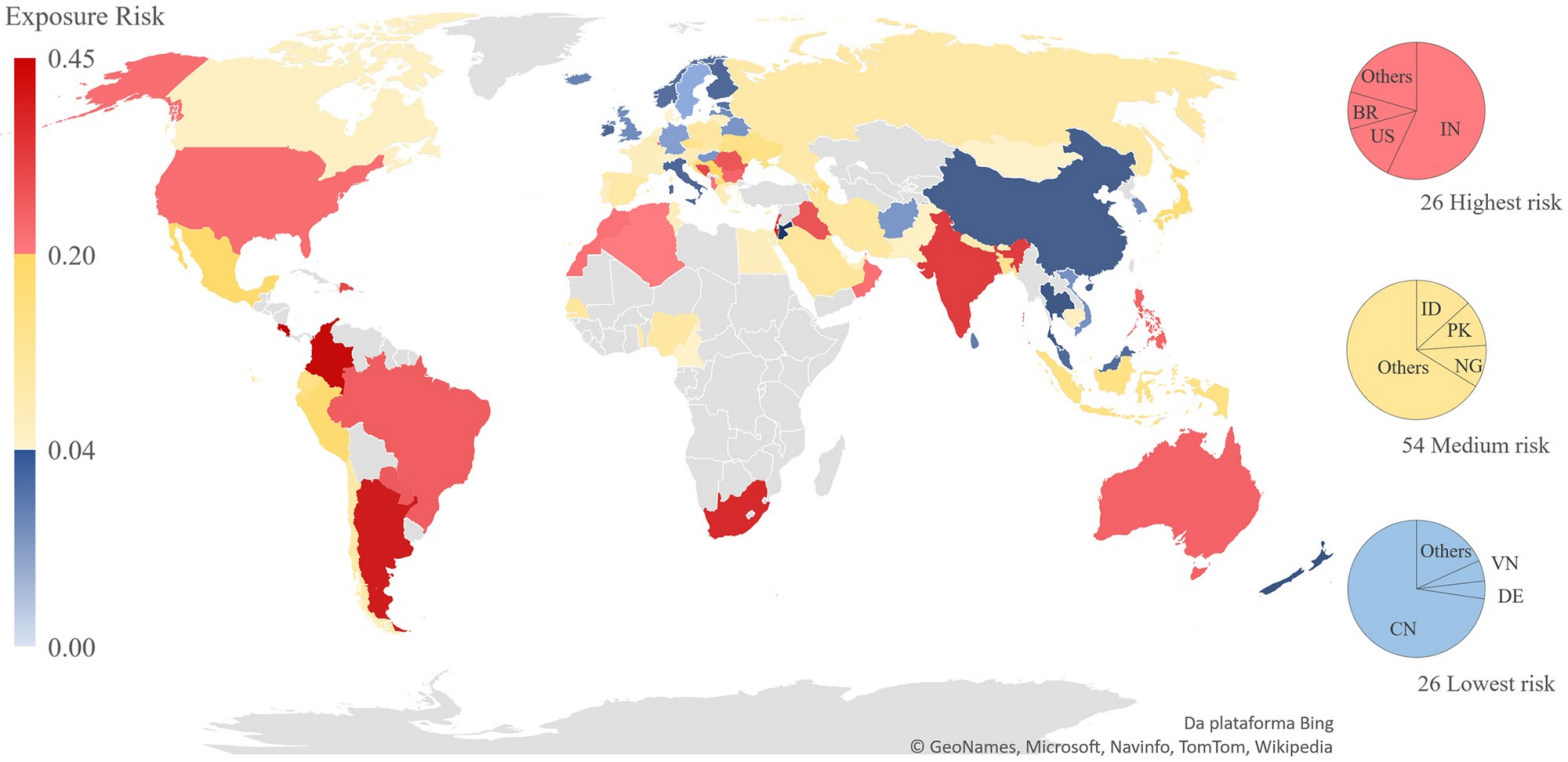
Exposure risk to COVID-19 by cluster of countries as of August 5, 2020. Risk ranging from zero (low) to one (high), based on incidence per country and speed. Colored 106 countries with first case reported before March 11, 2020 (84% of world population). In grey countries with first case reported after this date. Pie charts showing the population per country exposed to risk in each cluster. Microsoft product screenshots reprinted with permission from Microsoft Corporation.

India, the U.S., and Brazil were the countries most affected due to exposure risk to COVID-19 (0.308, 0.212, 0.259, respectively); with high speed (39.5%, 19.7%, 24.4%), moderate-to-high incidence (139.7, 1449.9, 1327.6 cases per 100000 ha), and the highest potentially exposed populations (Fig 3).

Cluster number 1 had Sint Maarten, Colombia, and Costa Rica with highest risk (0.445, 0.382, and 0.381, respectively) but also with a smaller population. Cluster number 3 had Brunei Darussalam, San Marino and Holy See with lowest exposure risk to COVID-19. However, China in Asia (0.011) and Germany in Europe (0.039) each have larger populations. Finally, New Zealand in Oceania was the third selected lower-risk country (0.007).

### Vulnerabilities of countries with high exposure risk to COVID-19

**Fig 4:**
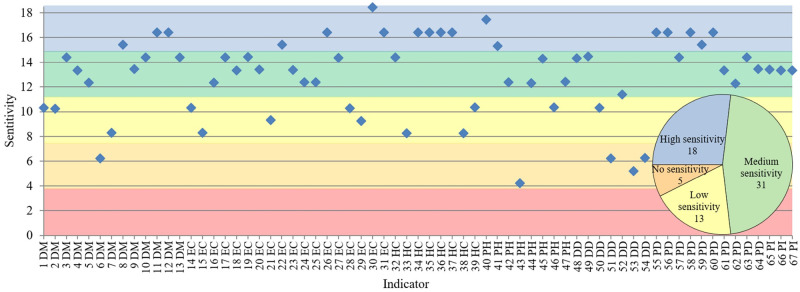
Indicator sensitivity to the pandemic control in Brazil, U.S., India, China, Germany, and New Zealand. Sensitivity could range from 0 (inverse compliance with the risk) to 18 (total compliance with the risk). The horizontal axis is labeled with each indicator sensitivity. Each colored area demonstrates a cluster of indicator sensitivity. The pie chart shows the total of indicators by the sensitivity cluster.

**Fig 5:**
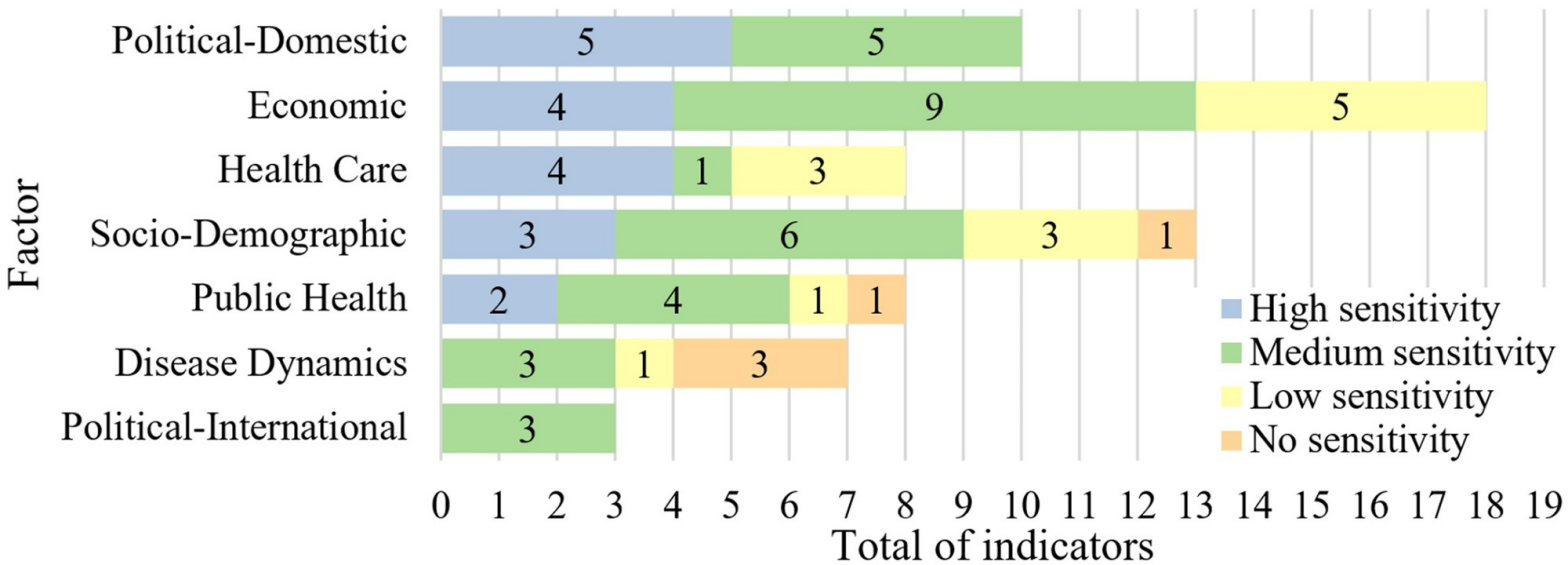
Indicator sensitivity by factor in Brazil, U.S., India, China, Germany and New Zealand. The bars show the total of indicators per sensitivity cluster for each vulnerability factor.

The comparative analysis among the highest and the lowest risk groups in different continents identified four clusters exhibiting vulnerabilities to pandemic control. Furthermore, 49 indicators showed medium-to-high sensitivity (Figs 4 and 5). All data is found in the S2 File.

The high sensitivity cluster had 18 indicators, seven of socioeconomic conditions (socio-demographic and economic), six of health infrastructure (public health and healthcare), and five of political-domestic factors (Table 1). The most discriminating vulnerability from India, the U.S., and Brazil to China, Germany, and New Zealand was the merchandise trade (sensitivity of 18.44). All indicators for the highest-risk group were below those of the lowest-risk group (31, 21, 23, 33, 72, and 40% of GDP, respectively). The second most discriminating vulnerability was immunization (90, 92, 84, 99, 97, and 92% of children age 12–23 months, respectively).

**Table 1:** High indicators sensitivity cluster.

	Indicator	BR	US	IN	CN	DE	NZ	S
30EC	Merchandise trade (of GDP) (*)	0.04	0	0.20	0.24	1.00	0.37	18.44
40PH	Immunization, measles (of children ages 12–23 months)	0	0.53	0.40	1.00	0.87	0.53	17.45
26EC	Mobile cellular subscriptions (per person)	0.25	0.88	0	0.60	0.88	1.00	16.44
60PD	Public Services (Fragile States Index) (*)	0	0.95	0.07	0.35	0.98	1.00	16.43
37HC	Maternal mortality ratio (per live births) (*)	0.62	0.92	0	0.84	1.00	0.97	16.43
12DM	Life expectancy at birth (*)	0.50	0.75	0	0.57	0.93	1.00	16.43
35HC	Mortality rate, infant (per live births)	0.64	0.91	0	0.84	1.00	0.94	16.42
34HC	Hospital beds (per population)	0.23	0.31	0	0.47	1.00	0.30	16.42
31EC	GINI index (*)	0	0.57	0.73	0.70	1.00	0.80	16.42
36HC	Mortality rate, under-5 (per live births) (*)	0.70	0.91	0	0.85	1.00	0.94	16.42
55PD	Governance (Government Effectiveness Index)	0	0.97	0.48	0.58	0.99	1.00	16.41
58PD	Governance (Political Stability and Absence of Violence Index)	0.20	0.56	0	0.26	0.62	1.00	16.41
11DM	Adolescent fertility rate (births per women ages 15–19) (*)	0	0.78	0.92	1.00	1.00	0.78	16.41
56PD	Governance (Regulatory Quality Index)	0	0.89	0.11	0.14	0.93	1.00	16.41
8DM	Population median age (years) (*)	0.20	0.58	0	0.58	1.00	0.55	15.44
59PD	Transparency International Corruption Perceptions Index	0	0.65	0.12	0.12	0.87	1.00	15.42
22EC	Industry, value added (of GDP) (*)	0	0	0.36	1.00	0.41	0.09	15.41
41PH	Water supply	0.75	0.90	0	0.02	1.00	1.00	15.34

The vulnerabilities were identified based on each indicator measurement (ranging from 0 [bad] to 1 [good]). BR = Brazil. US = United States of America. IN = India. CN = China. DE = Germany. NZ = New Zealand. S = Indicator Sensitivity.

(*) = New indicators.

The medium sensitivity cluster had 31 indicators (Table 2), with six of health infrastructure (four of public health, one of healthcare, and three of disease dynamics factors); 15 of socioeconomic conditions (six of socio-demographic and nine of economic); and eight political factors (five domestic and three international).

**Table 2:** Medium indicators sensitivity cluster.

	Indicator	BR	US	IN	CN	DE	NZ	S
49DD	Annual average temperature	0	0.79	0.02	0.71	1.00	0.75	14.46
19EC	Infrastructure and technology: transportation (logistics)	0	0.74	0.16	0.51	1.00	0.74	14.43
17EC	Inflation, GDP deflator annual (*)	0.30	0.50	0	0.25	0.70	1.00	14.42
3DM	Average annual population growth rate	0.39	0.44	0	0.79	1.00	0.19	14.42
63PD	Government stability (Fragility Index)	0.50	0.70	0	0.40	1.00	0.90	14.41
10DM	Fertility rate, total (births per woman) (*)	0.83	0.83	0	0.83	1.00	0.83	14.40
13DM	Human Development Index (*)	0.39	0.93	0	0.38	1.00	0.94	14.40
32HC	Skilled health professionals: density—latest available year	0.42	0.58	0	0.21	1.00	0.75	14.40
57PD	Governance Indicators Rule of Law Index	0	0.84	0.21	0.07	0.87	1.00	14.39
27EC	Individuals using the Internet (of population)	0.64	0.94	0	0.35	0.98	1.00	14.38
48DD	Average rainfall per year (mm per year))	0	0.94	0.61	1.00	0.95	0.03	14.34
45 PH	Domestic General Government Health Expenditure % GDP (*)	0.33	0.89	0	0.26	1.00		14.30
64PD	Human rights (Amnesty International Political Terror Scale)	0	0.33	0.33	0	1.00	1.00	13.43
9DM	Income share held by lowest 20% (*)	0	0.44		0.76	1.00		13.43
20EC	Infrastructure and technology: transportation (paved roads)	0	0.61	0.44	0.40	1.00	0.61	13.40
65PI	Net Official Development Assistance (per capita)	0		0.09	1.00			13.39
23EC	Index of Technological Sophistication	0		0.33	1.00			13.37
61PD	State legitimacy (Fragile States Index) (*)	0.25	0.71	0.58	0	1.00	1.00	13.35
66PI	Net Official Development Assistance received	0.70		0	1.00			13.33
67PI	Net financial flows, multilateral (NFL) (*)	0.74		0	1.00			13.33
4DM	Literacy rate—population aged 15 and over	0	0.82		1.00			13.32
18EC	Poverty headcount ratio at $1.90 a day (of population)	0.84		0	1.00			13.32
47PH	Country leading or contributing to GHSA action package	0	1.00	0	1.00	1.00	0	12.40
42PH	Sanitation	0.71	1.00	0	0.62	0.98	1.00	12.39
24EC	High-technology exports (of manufactured exports) (*)	0.18	0.45	0	1.00	0.32	0.05	12.37
25EC	Investments in research and development (*)	0.31	0.94	0	0.63	1.00	0.27	12.36
5DM	Gross enrollment rate all school levels (*)	0.59	0.82	0	0.21	0.76	1.00	12.36
16EC	GDP growth (annual) (*)	0	0.35	0.87	1.00	0.04	0.45	12.33
44PH	UHC index of essential service coverage (*)	0.87	1.00	0	0.83	0.96		12.29
62PD	Democracy (Polity IV Project Democracy Index)	0.80	0.80	0.90	0	1.00	1.00	12.25
52DD	Ecological: Global deforestation rates (of land area)	0.32	0	1.00	0.84	0.85	0.32	11.37

The countries vulnerabilities with high exposure risk to COVID-19 were identified based on each indicator measurement (ranging from 0 (bad) to 1 (good)). BR = Brazil. US = United States of America. IN = India. CN = China. DE = Germany. NZ = New Zealand. S = Indicator Sensitivity.

(*) = New indicators.

There are some indicators without data to one or more countries from the original database. It was a limitation to classify the indicator sensitivity.

### The indicators sensibility compared to the IDVI sensibility

**Fig 6:**
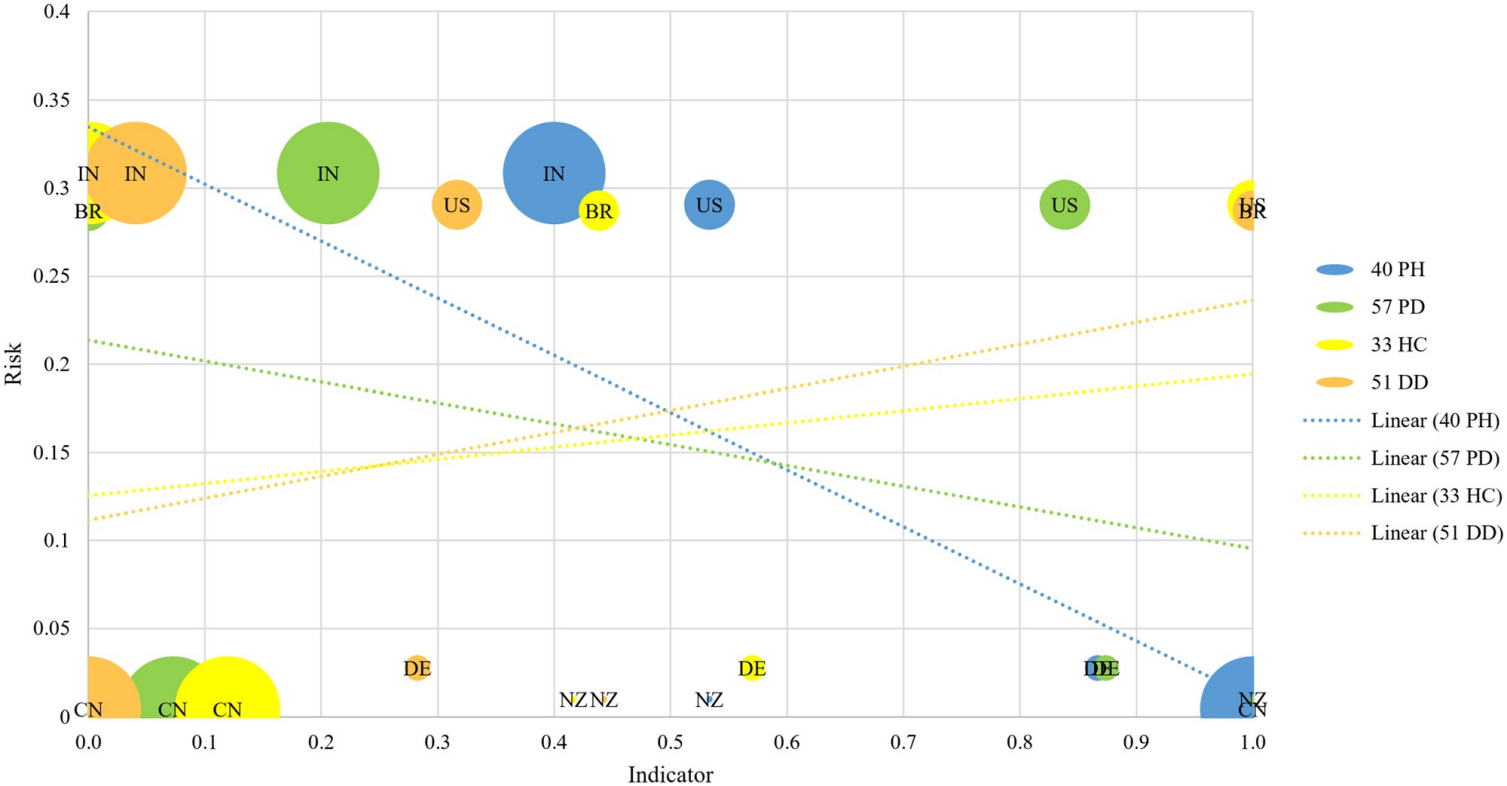
Exposure risk to COVID-19 as a function of each indicator. Risk: 0 (low) to 1 (high). Indicator: 0 (bad) to 1 (good). One example of indicator by each sensitivity cluster: Blue = High. Green = Medium. Yellow = Low. Orange = No. A good indicator for risk prediction focuses on countries that have fought the pandemic well in the lower right quadrant of the graph and countries that have fought poorly in the upper left quadrant. Factor type: PH = Public Health. PD = Political-Domestic. HC = Health Care. DD = Dinamic Disease. Trend line for each indicator (Most negative slope = most sensitive indicator. Most positive slope = least sensitive indicator). The area of circles is proportional to the country population. BR = Brazil. US = United States of America. IN = India. CN = China. DE = Germany. NZ = New Zealand.

**Fig 7:**
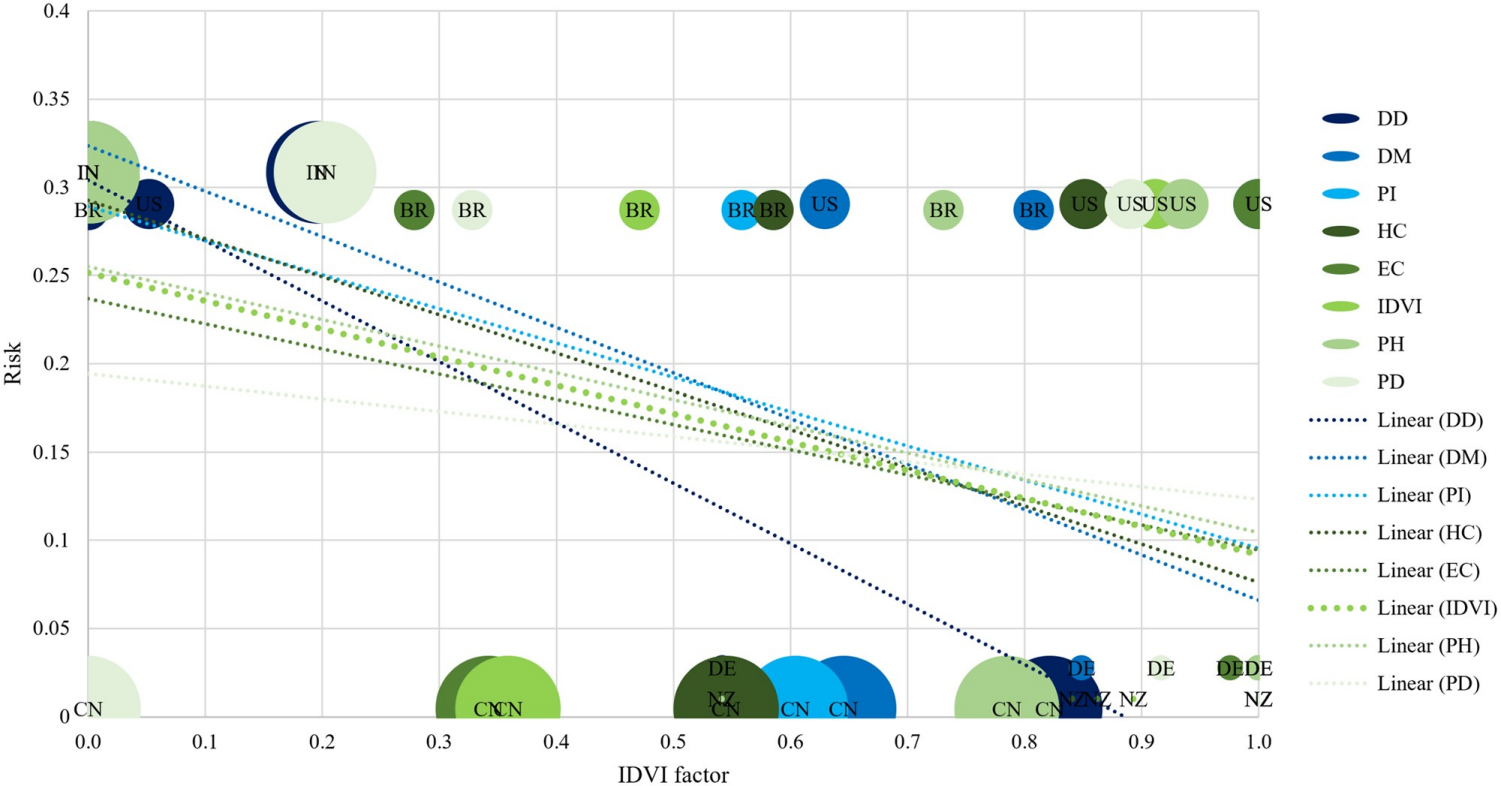
Exposure risk to COVID-19 as a function of IDVI factor published in 2016. Risk: 0 (low) to 1 (high). IDVI factor: 0 (bad) to 1 (good). The IDVI and IDVI factors by sensitivity cluster: Blue = High. Green = Medium. A good indicator for risk prediction focuses on countries that have fought the pandemic well in the lower right quadrant of the graph and countries that have fought poorly in the upper left quadrant. Factor type: PH = Public Health. PD = Political-Domestic. HC = Health Care. DD = Dinamic Disease. IDVI = Infectious Disease Vulnerability Index. Trend line for each indicator (Most negative slope = most sensitive indicator. Most positive slope = least sensitive indicator). The area of circles is proportional to the country population. BR = Brazil. US = United States of America. IN = India. CN = China. DE = Germany. NZ = New Zealand.

The indicators of each sensitivity cluster behave differently in relation to risk (Fig 6). We applied this methodology to IDVI, and all their factors displayed medium-to-high sensitivity without great distinction between them, see Fig 7, which did not seem so clear when looking at Table 3. Thus, these results agreed that the USA, Brazil, and India have similarities, and indicated that the Disease Dynamics factor has the highest sensitivity, but the doubt remained which indicator should be considered first. To that end, our individual analysis of the indicators, in fact, signaled high sensitivity in indicators of different factors that should be considered first to facing the Covid-19 Pandemic.

**Table 3:** The sensitivity of the IDVI factors published in 2016.

	BR	US	IN	CN	DE	NZ	S	Sensitivity
Disease Dynamics	0	0.05	0.20	0.82	0.54	1.00	18.51	High
Demographic	0.81	0.63	0	0.65	0.85	1.00	16.41	High
Political-International	0.56	1.00	0	0.60	1.00	1.00	15.40	High
Health Care	0.59	0.85	0	0.54	1.00	0.84	12.39	Medium
Economic	0.28	1.00	0	0.34	0.98	0.86	12.39	Medium
Public Health	0.73	0.94	0	0.78	1.00	0.54	12.37	Medium
Political-Domestic	0.33	0.89	0.20	0	0.92	1.00	12.35	Medium

The countries vulnerabilities with high exposure risk to COVID-19 were identified based on each IDVI factor measurement (ranging from 0 (bad) to 1 (good)). BR = Brazil. US = United States of America. IN = India. CN = China. DE = Germany. NZ = New Zealand. S = Sensitivity.

### Response capacity of each country

The response capacity of each country is directly related to some specific indicators of socioeconomic, health infrastructure, and political factors, suggesting priority attention from India, Brazil, and the U.S. to indicators with high sensitivity.

**Fig 8:**
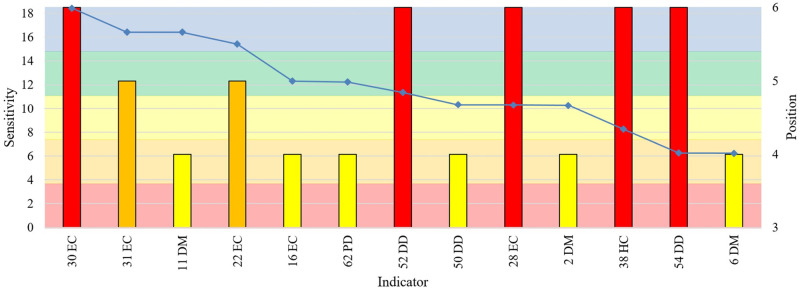
Indicators in which U.S. ranked fourth or above. Indicator Sensitivity (blue points), values could range from 0 (inverse compliance with the risk) to 18 (total compliance with the risk). The horizontal axis labeled with each indicator. Relevant Position of the Group to Indicator (columns, colored according to position). Indicators were ordered according to sensitivity (highest first).

The U.S. had high sensitivity in socio-demographic factor (adolescent fertility rate) and economic indicators: merchandise trade, the GINI index, and industry. In the political–domestic factor, the democracy index had a medium sensitivity, with the U.S. and Brazil tied (Fig 8).

**Fig 9:**
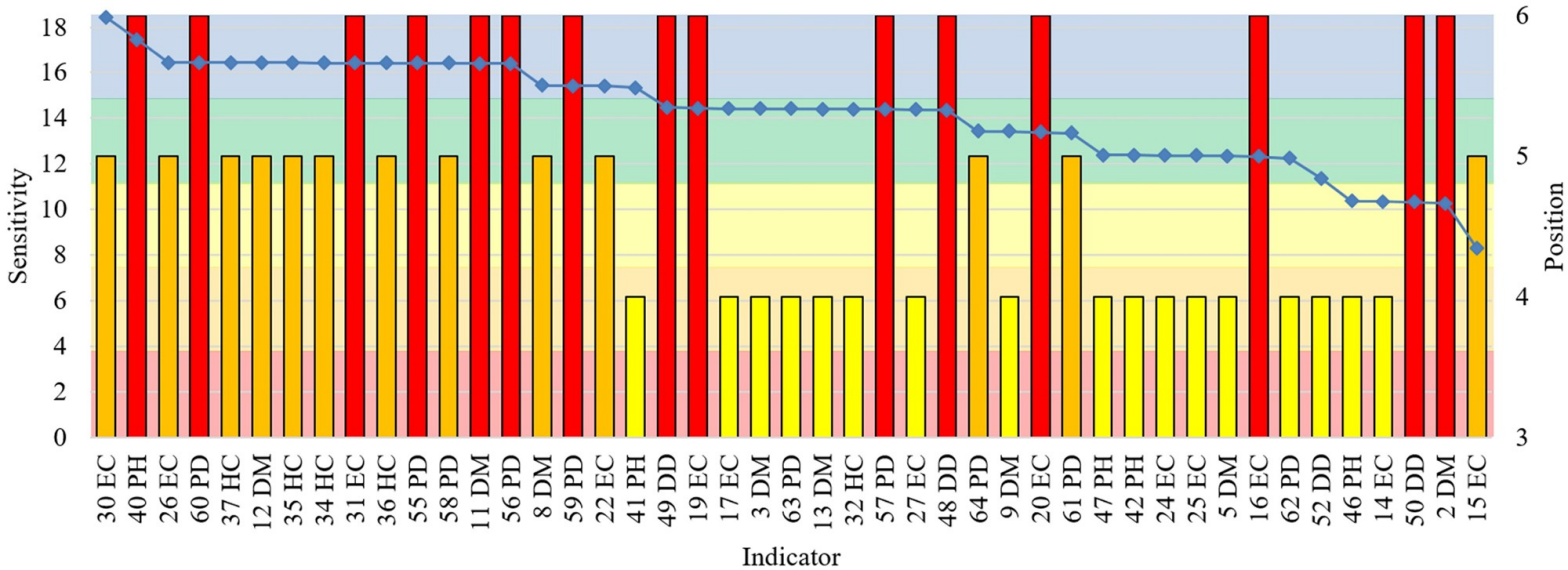
Indicators in which Brazil ranked fourth or above. Indicator Sensitivity (blue points), values could range from 0 (inverse compliance with the risk) to 18 (total compliance with the risk). The horizontal axis labeled with each indicator. Relevant Position of the Group to Indicator (columns, colored according to position). Indicators were ordered according to sensitivity (highest first).

Brazil should be considered at the first: political–domestic (public services, governance), economic (merchandise trade, mobile cellular subscriptions, GINI, and industry), healthcare (hospital beds and mortalities- maternal, infant, under-5), public health (immunization), and socio-demographic (adolescent fertility rate, life expectancy at birth and population median age). Within the medium sensitivity range, Brazil had should be careful too with infrastructure and technology (transportation: logistics and roads paved), average temperature, and GDP growth (Fig 9).

**Fig 10:**
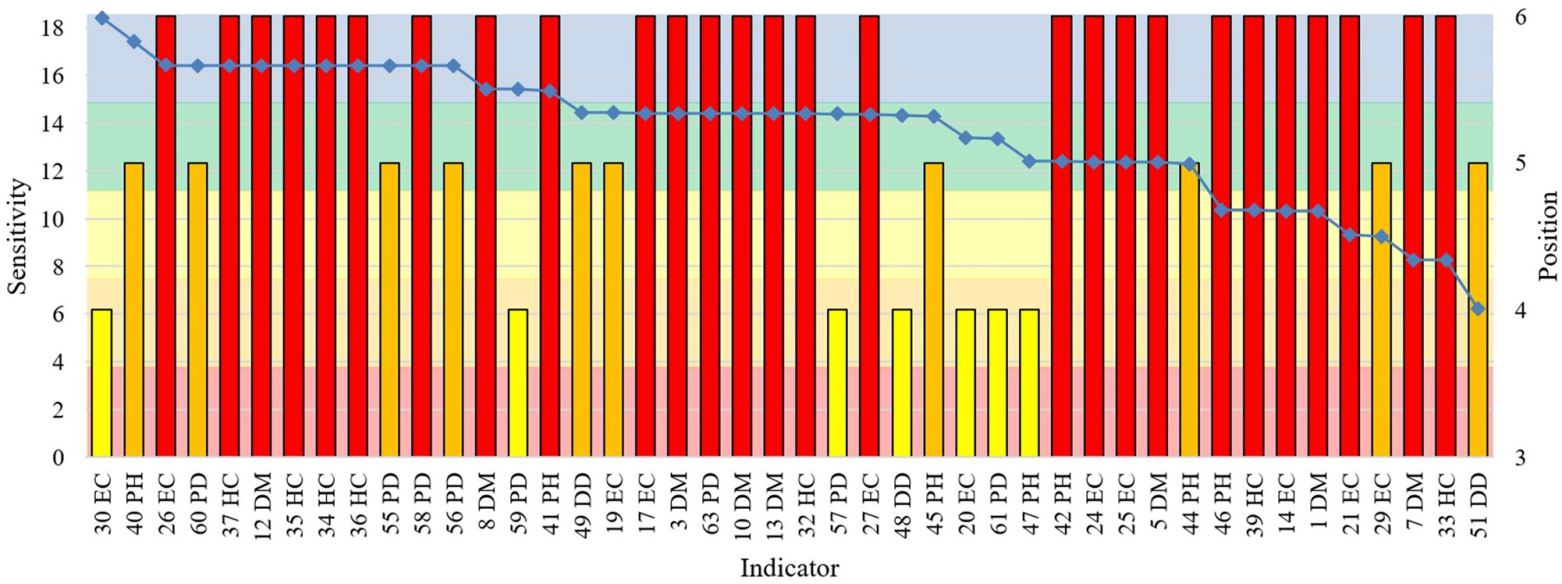
Indicators in which India ranked fourth or above. Indicator Sensitivity (blue points), values could range from 0 (inverse compliance with the risk) to 18 (total compliance with the risk). The horizontal axis labeled with each indicator. Relevant Position of the Group to Indicator (columns, colored according to position). Indicators were ordered according to sensitivity (highest first).

India had vulnerabilities with high sensitivity in merchandise trade and mobile cellular subscriptions (economic), life expectancy at birth and population median age (socio-demographic), but should be considered at the first: healthcare factors (mortalities, water supply, hospital beds), political–domestic (public services, governance), and public health (immunization) (Fig 10).

## Discussion

### Added value of this study

There are some studies on exposure risk related to Covid-19, but they are of various types and have different objectives and methods than this study. Some check the risk to determine the psychological consequences, impacts on the economy, or risk factors of health. Others estimate future events.

For example, COSRE [17] is a system that estimates the social risk of the community. The risk of exposure was defined as the likelihood that people would encounter potential cases in different public places.

Another study [18], with a similar objective to this study, developing a planning tool that compares the estimated general vulnerability, with confirmed cases of COVID-19 reported in real-time. The vulnerability considered new variables (indicators) grouped into categories (factors), as in the reference articles cited in the introduction, but using estimates to replace missing data from various sectors, at the county (local) level.

Among the advantages of our study, we can say that it is not a probability, but a risk of real exposure. We use historical and current figures for all countries with registered cases, for their performance in combating the pandemic (incidence, speed) and population, allowing the calculation of risk, without the need for data on the behavior of the population, habits and local public policies of combat the pandemic, to make it possible to assess the performance of one country (or group of countries) in relation to others. In this study, the impacts of the pandemic can also be explored through spatio-temporal analysis and assess changes in risk as the pandemic develops.

On the other hand, this number is very dependent on the momentary performance of the region analyzed in combating Covid-19, being inadequate to assess this performance a posteriori, when speed is no longer a determining factor, making it more simple, or can be adapted.

The risk made it possible to identify the real performance of nations in combating Covid-19 and analyze the poor variables. In other words, based on the risk of exposure, we created the model to assess the countries’ response capacity.

### Actors involved in pandemic control

These results can be used in a practical way by people, health systems, and policymakers, to the development of actions.

The vulnerability assessment model can be used to analyze more countries or new data over time or adapted to local circumstances to prioritize public policy actions.

Researchers can carry out specific studies to further analyze these indicators or improve the method.

Policymakers can use the results in their risk management tools and action plans, in relation to the current government’s economy, local health system, attitude, and policy.

Health systems will be able to make more adequate forecasts, considering that patient behavior and external factors can greatly influence the effects of a pandemic.

People will be able to better understand their role as a community, which can influence the behavior of others and improve institutions at the local level and the results of a country.

The risk of exposure can be reduced when interconnected actions are carried out concurrently to achieve better indicators, in a joint effort between all actors, public and private.

### Interrelated actions

The greatest vulnerability within the highest-risk group (the U.S., Brazil, and India) was the merchandise trade, related to the economic factor, highlighting the world dependence on Chinese trade, such as the production of protective materials, equipment, and diagnostic tests. People can contribute with the indicator, by prioritizing consumption of local products, cooperatives, and new initiatives; health systems promoting national industrial development and production; public policymakers with alternatives for market balance, trade protection measures and flow from the production chain; for example.

The second vulnerability was immunization, indicating the difficulty these countries’ public health systems will face when it is possible to immunize the population and thereby bring the pandemic under some measure of control. People can search and share only reliable information, trust health authorities, and comply with vaccination schedules and campaigns. Health systems must expand access, evaluate results, conduct an active search, and promote stimulation and promotion of science, technology and innovation of national inputs and products. Public policymakers must ensure vaccination in the population as a whole; among others.

As expected, the socioeconomic indicators included in IDVI are related to each country’s response capacity to the virus, such as mobile cellular subscriptions, infrastructure and technology (logistics), percentage of the population using the internet, index of technological sophistication, and poverty headcount. These indicators can be considered as well: merchandise trade, GINI index, percentage of industry value added in the GDP, life expectancy at birth, adolescent fertility rate, inflation, Human Development Index, income share held by the lowest 20%, high-technology exports, investments in research and development, gross enrollment rate in all school levels, and GDP growth. Therefore, social determinants as physical environment can have a considerable effect on COVID-19 outcomes [19].

In the same way, we observed public health indicators selected by the IDVI, such as immunization, water supply, and sanitation facilities, but these can also be incorporated: Domestic General Government Health Expenditure and UHC index of essential service coverage. Related to infrastructure too are the indicators of the healthcare factors included in the IDVI such as hospital beds, skilled health professionals density, and infant mortality rate. Maternal mortality ratio and under-5 mortality rate were tested too and had the same results, so they can help to identify vulnerabilities as well.

Disease–dynamics indicators such as deforestation rates, average rainfall, and temperature had medium sensitivity and were associated with the exposure risk to COVID-19, as in previous studies [20–22].

The indicator of gross national income included in the political–international factor of the IDVI displayed medium sensitivity. Indicators of the political–domestic factors related by the IDVI had medium-to-high sensitivity, such as governance (rule of law, effectiveness, regulatory quality, and political stability); the transparency/corruption perceptions index; the government stability fragility index; and democracy. In addition, we added fragile states in public services and state legitimacy with respectively high and medium sensitivity.

### Countries actions

India had the highest exposure risk to COVID-19, which is mainly related to socio-demographic vulnerabilities and healthcare. The actions of all actors in relation to these indicators deserve to be the objective of a specific detailed study, which is not in the scope of this one. Countries with higher levels of extreme poverty, such as India [23], have difficulties in accessing drinking water and other basic healthcare. Due to such vulnerabilities, the country was unable to maintain the strict lockdown measures initially implemented by the government. The analysis of these specific indicators may allow better decision-making until a vaccine is accessible to the entire population.

Despite the U.S. being a high-income country, it has had a low capacity of response and control throughout the COVID-19 pandemic. The U.S. and Brazil reported increasing numbers of cases, heralding an imminent wave of fatalities since March 2020, which has brought with it many problems related to political factors [24–28]. The pandemic has aggravated political problems, and these have harmed the health of the population in those countries. People need to better exercise their role in democracy, for example, by supporting only real information and authorities committed to technical and scientific knowledge. Health systems and policymakers, on the other hand, can reduce political interference and guarantee technical actors in decision-making.

A curiosity is that the U.S. and Brazil had the same industry indicator in% of GDP, with high sensitivity. Health systems and public policymakers in these countries may need to make an assessment of the industrial policy adopted, especially in the area of health, and people may collaborate with active participation in the environments of articulation.

The GINI index, which is an instrument to measure the degree of income concentration in a given group, also makes up the economic factor with high sensitivity related to the spread of the virus, to be evaluated by the actors in Brazil and the U.S. People can collaborate by offering services online, remotely and purchasing home products and from small producers. Health systems need to ensure equity in care, efficiency, effectiveness, and effectiveness in the conscious use of resources. Policymakers should increase resource allocation to reduce inequities, rather than exacerbating them [23].

For Brazil, it is still necessary to evaluate what the actors can do to reduce the adolescent fertility rate, which proved to be very expressive. People must face this situation and discuss the topic of teenage pregnancy. Health systems and policymakers must take more assertive and transversal actions with sectors such as education.

China had demonstrated a better response capacity than the U.S., despite having the lowest indicators. For example, China has taken better control of the pandemic, with fewer COVID-19 cases and deaths. China was the epicenter of the pandemic; therefore, it learned to deal with the situation and quickly adopted different protective measures [4, 8, 29–33].

In addition, Germany has conducted a lot of testing, interventions [33, 34], and has had one of the lowest speeds of incidence of cases among countries in Europe, regardless of having the largest population on the continent. New Zealand, in Oceania, has displayed a great control capacity, eliminating the curve of cases instead of flattening it [35, 36].

## Conclusions

The exposure risk to COVID-19 in different countries revealed internal vulnerabilities, providing information to policymakers in order to increase the response capacity of countries in areas such as health infrastructure, socioeconomics, and politics to effectively enhance these countries’ control capacities.

The outcomes showed that indicators should also be considered individually to respond to each health condition, based on the specific risks for each country.

Furthermore, IDVI ranked the United States among the top eight countries (188 out of 195) in 2016 because its methodology results in the average of different factors with various indicators. However, we can conclude that these factors should not be limited to one unique approach to risk estimation. This approach is insufficient to predict the difficulty of some high-income countries in curbing the proliferation of the virus, but their indicators can indicate the way. Schwalbe and colleagues emphasized the necessity to move from a one-size-fits-all approach to one that focuses on the most at-risk populations [37]. They also explained that the risk factors need to be understood, including the effects and their interaction, and that countries must be willing to improve strategies to help to dispel the popular misconception that everyone is at the same risk.

The equity between individuals must be considered in times of pandemic because these countries have particularities in their social classes and regions that make them more vulnerable to the spread and lethality of the disease. In addition, lethality is correlated with the healthcare burden. Misinformation and poor communication disproportionally affect individuals with less access to information channels. These people are therefore more likely to ignore government health warnings [23].

The indicators verified individually are not the causes of the spread, but they might represent the effect that this spread would have on the population. In other words, the indicators can signal the protection focus to which resources should be directed, according to the reality of each country.

The analysis between the indicators of several countries allows the achievement of impressive correlations, since economic development is relative between countries, as the Theory of Economic Complexity demonstrates.

Only socioeconomic and health infrastructure indicators are not enough to control the pandemic. Political factors are as important to effectively guide the population. Domestic–political factors had greater weight in indicator measurements with high sensitivity, followed by healthcare, indicating that the U.S., Brazil, and India have to guide, coordinate, and motivate the general population and vulnerable groups especially to comply with measures such as isolation and social distancing.

Many countries still face a lack of tests, drugs, hospital equipment, and personal protective equipment. These difficulties are related to the high sensitivity identified by indicators related to merchandise trade, industry, technology, and politics, signaling the need to improve contact networks, technological measures, and productive self-sufficiency, in order to support these countries’ healthcare systems in combating outbreaks of infectious diseases and in the management of health emergencies.

We conclude that countries of similar size (like the U.S., Brazil, India, China, and Germany) have had different results in controlling the pandemic because they have different vulnerabilities, which have influenced the way people and their governments behave.

This study of the vulnerability assessment is accurate, for decision making in the real world. It is not estimation. The exposure risk was defined to analyze the country as a whole, disregarding any internal regional differences, but it can also be used and adapted to local circumstances. It is possible to calculate the exposure risk using incidence, speed and population of states, regions, or county-level to compare the situation in real-time and to identify the weaknesses and prioritize public policy actions to focus on your indicators more sensitive related to infectious disease. On a similar note, this model might be expanded with other techniques, updated systematically with new data of the evolution of the disease, and used to analyze more countries to contain the pandemic.

## Supporting information

S1 File COVID-19-geographic-distribution-worldwide, incidence, speed population, exposure risk. (PDF)

S2 File Indicators sensitivity. (PDF)
