# Supplementary material for: The exposure risk to COVID-19 in most affected countries: A vulnerability assessment model
Source: PLoS One. 2021 Mar 4;16(3):e0248075. doi: 10.1371/journal.pone.0248075 (PMC7932136; doi:10.1371/journal.pone.0248075)
Supplement: S1 File — (PDF) [file pone.0248075.s001.pdf]

S1 COVID-19 - Geographic-disbtribution-worldwide _ incidence speed population exposure risk

	Min. dateRep	Max. popData2019	Cumulative cases at 05-10-2020	% increase incidence last 15 days.
Afghanistan	31/12/2019	38041757	36829	0,032963154
Albania	09/03/2020	2862427	5750	0,274608696
Algeria	31/12/2019	43053054	32504	0,27113586
Andorra	03/03/2020	76177	939	0,05857295
Angola	22/03/2020	31825299	1164	0,35652921
Anguilla	27/03/2020	14872	3	0
Antigua_and_Barbuda	15/03/2020	97115	92	0,173913043
Argentina	04/03/2020	44780675	206730	0,386920137
Armenia	31/12/2019	2957728	39298	0,102906
Aruba	13/03/2020	106310	132	0,128787879
Australia	31/12/2019	25203200	18729	0,35559827
Austria	31/12/2019	8858775	21472	0,083504098
Azerbaijan	31/12/2019	10047719	32910	0,152537223
Bahamas	16/03/2020	389486	715	0,756643357
Bahrain	31/12/2019	1641164	42132	0,123326688
Bangladesh	09/03/2020	163046173	244020	0,149852471
Barbados	18/03/2020	287021	132	0,196969697
Belarus	31/12/2019	9452409	68250	0,029846154
Belgium	31/12/2019	11455519	72338	0,102463436
Belize	24/03/2020	390351	72	0,416666667
Benin	17/03/2020	11801151	1914	0,163009404
Bermuda	20/03/2020	62508	157	0,025477707
Bhutan	06/03/2020	763094	105	0,142857143
Bolivia	12/03/2020	11513102	83361	0,268350907
Bonaire, Saint Eustatius and Saba	02/04/2020	25983	13	0,230769231
Bosnia_and_Herzegovina	06/03/2020	3300998	12858	0,331855654
Botswana	01/04/2020	2303703	804	0,350746269
Brazil	31/12/2019	211049519	2801921	0,243859481
British_Virgin_Islands	27/03/2020	30033	9	0,111111111
Brunei_Darussalam	10/03/2020	433296	141	0
Bulgaria	08/03/2020	7000039	12159	0,265646846
Burkina_Faso	11/03/2020	20321383	1156	0,078719723
Burundi	01/04/2020	11530577	395	0,184810127
Cambodia	31/12/2019	16486542	243	0,189300412
Cameroon	07/03/2020	25876387	17718	0,088102495
Canada	31/12/2019	37411038	117777	0,056581506
Cape_Verde	21/03/2020	549936	2631	0,212846826
Cases_on_an_international_conveyance_Japan	31/12/2019		0	0
Cayman_Islands	20/03/2020	64948	416	0,014084507
Central_African_Republic	16/03/2020	4745179	4618	0,015158077
Chad	20/03/2020	15946882	938	0,052238806
Chile	04/03/2020	18952035	362962	0,082468688
China	31/12/2019	1433783692	88206	0,028444777
Colombia	07/03/2020	50339443	334979	0,390991674
Comoros	02/05/2020	850891	388	0,139175258
Congo	16/03/2020	5380504	3546	0,195995488
Costa_Rica	07/03/2020	5047561	19837	0,418561274
Cote_dIvoire	12/03/2020	25716554	16293	0,121585957
Croatia	31/12/2019	4076246	5318	0,178262505

	Min. dateRep	Max. popData2019	Cumulative cases at 05-10-2020	% increase incidence last 15 days.
Cuba	12/03/2020	11333484	2701	0,094409478
CuraÃ§ao	13/03/2020	163423	29	0,034482759
Cyprus	10/03/2020	875899	1180	0,120338983
Czechia	31/12/2019	10649800	17286	0,184426704
Democratic_Republic_of_the_Congo	11/03/2020	86790568	9177	0,080091533
Denmark	31/12/2019	5806081	14073	0,057628082
Djibouti	19/03/2020	973557	5248	0,043445122
Dominica	23/03/2020	71808	18	0
Dominican_Republic	31/12/2019	10738957	74295	0,273760011
Ecuador	31/12/2019	17373657	87963	0,151688778
Egypt	31/12/2019	100388076	94752	0,067017055
El_Salvador	19/03/2020	6453550	18262	0,331562808
Equatorial_Guinea	15/03/2020	1355982	4821	0,362995229
Eritrea	22/03/2020	3497117	282	0,109929078
Estonia	31/12/2019	1324820	2091	0,033476805
Eswatini	15/03/2020	1148133	2856	0,360644258
Ethiopia	14/03/2020	112078727	19877	0,486491925
Falkland_Islands_(Malvinas)	04/04/2020	3372	13	0
Faroe_Islands	06/03/2020	48677	227	0,158590308
Fiji	20/03/2020	889955	27	0,037037037
Finland	31/12/2019	5517919	7483	0,019109983
France	31/12/2019	67012883	192334	0,081004919
French_Polynesia	19/03/2020	279285	62	0
Gabon	13/03/2020	2172578	7646	0,158645043
Gambia	18/03/2020	2347696	671	0,833084948
Georgia	31/12/2019	3996762	1197	0,131996658
Germany	31/12/2019	83019213	212022	0,04564149
Ghana	13/03/2020	30417858	37812	0,248122289
Gibraltar	20/03/2020	33706	189	0,047619048
Greece	31/12/2019	10724599	4855	0,173635427
Greenland	20/03/2020	56660	14	0,071428571
Grenada	23/03/2020	112002	24	0,041666667
Guam	19/03/2020	167295	389	0,159383033
Guatemala	15/03/2020	17581476	52365	0,254482956
Guernsey	20/03/2020	64468	252	0
Guinea	14/03/2020	12771246	7489	0,120042729
Guinea_Bissau	27/03/2020	1920917	2032	0,040354331
Guyana	13/03/2020	782775	497	0,32193159
Haiti	20/03/2020	11263079	7532	0,062267658
Holy_See	07/03/2020	815	12	0
Honduras	12/03/2020	9746115	44299	0,218695682
Hungary	05/03/2020	9772756	4553	0,045244893
Iceland	31/12/2019	356991	1918	0,041188738
India	31/12/2019	1366417756	1908254	0,394634572
Indonesia	31/12/2019	270625567	115056	0,233295091
Iran	31/12/2019	82913893	314786	0,12257216
Iraq	31/12/2019	39309789	134722	0,297122964
Ireland	31/12/2019	4904240	26253	0,018550261
Isle_of_Man	21/03/2020	84589	336	0
Israel	31/12/2019	8519373	76642	0,325748284
Italy	31/12/2019	60359546	248419	0,015276609

	Min. dateRep	Max. popData2019	Cumulative cases at 05-10-2020	% increase incidence last 15 days.
Jamaica	12/03/2020	2948277	920	0,120652174
Japan	31/12/2019	126860299	41129	0,37426147
Jersey	20/03/2020	107796	343	0,034985423
Jordan	03/03/2020	10101697	1224	0,000816993
Kazakhstan	15/03/2020	18551428	102936	0,286274967
Kenya	14/03/2020	52573967	23202	0,40647358
Kosovo	14/03/2020	1798506	9274	0,366292862
Kuwait	31/12/2019	4207077	68774	0,131023352
Kyrgyzstan	19/03/2020	6415851	38110	0,258698504
Laos	25/03/2020	7169456	20	0,05
Latvia	03/03/2020	1919968	1249	0,045636509
Lebanon	31/12/2019	6855709	5062	0,42611616
Lesotho	15/05/2020	2125267	726	0,505509642
Liberia	17/03/2020	4937374	1216	0,089638158
Libya	25/03/2020	6777453	4063	0,517597834
Liechtenstein	05/03/2020	38378	89	0,02247191
Lithuania	31/12/2019	2794184	2137	0,088909686
Luxembourg	31/12/2019	613894	6917	0,189677606
Madagascar	21/03/2020	26969306	11895	0,398654897
Malawi	03/04/2020	18628749	4347	0,299516908
Malaysia	31/12/2019	31949789	9002	0,022439458
Maldives	08/03/2020	530957	4446	0,332883491
Mali	26/03/2020	19658023	2543	0,026740071
Malta	07/03/2020	493559	799	0,155193992
Mauritania	15/03/2020	4525698	6382	0,071921028
Mauritius	20/03/2020	1269670	344	0,002906977
Mexico	31/12/2019	127575529	502966	0,197102786
Moldova	08/03/2020	4043258	25814	0,182033005
Monaco	31/12/2019	33085	87	0,126436782
Mongolia	10/03/2020	3225166	293	0,020477816
Montenegro	18/03/2020	622182	3361	0,446295745
Montserrat	21/03/2020	4991	13	0,076923077
Morocco	03/03/2020	36471766	27217	0,354741522
Mozambique	23/03/2020	30366043	2029	0,257269591
Myanmar	17/03/2020	54045422	356	0,042134831
Namibia	15/03/2020	2494524	2470	0,455870445
Nepal	31/12/2019	28608715	21009	0,150649722
Netherlands	31/12/2019	17282163	55955	0,072290233
New_Caledonia	21/03/2020	282757	22	0
New_Zealand	31/12/2019	4783062	1219	0,011484824
Nicaragua	19/03/2020	6545503	3902	0,193490518
Niger	21/03/2020	23310719	1152	0,040798611
Nigeria	31/12/2019	200963603	44433	0,162221772
North_Macedonia	31/12/2019	2077132	11140	0,169748654
Northern_Mariana_Islands	31/03/2020	57213	46	0,173913043
Norway	31/12/2019	5328212	9333	0,032679739
Oman	31/12/2019	4974992	79159	0,13591632
Pakistan	31/12/2019	216565317	281136	0,05349724
Palestine	06/03/2020	4981422	16628	0,367332211
Panama	10/03/2020	4246440	69424	0,216034801
Papua_New_Guinea	21/03/2020	8776119	114	0,833333333

	Min. dateRep	Max. popData2019	Cumulative cases at 05-10-2020	% increase incidence last 15 days.
Paraguay	08/03/2020	7044639	5852	0,359535202
Peru	07/03/2020	32510462	439890	0,186885358
Philippines	31/12/2019	108116622	112593	0,388079188
Poland	04/03/2020	37972812	48149	0,161290993
Portugal	03/03/2020	10276617	51681	0,05630696
Puerto_Rico	28/03/2020	2933404	19324	0,355154212
Qatar	31/12/2019	2832071	111538	0,04035396
Romania	31/12/2019	19414458	55241	0,309588892
Russia	31/12/2019	145872260	861423	0,097439934
Rwanda	15/03/2020	12626938	2099	0,223916151
Saint_Kitts_and_Nevis	26/03/2020	52834	17	0
Saint_Lucia	15/03/2020	182795	25	0,08
Saint_Vincent_and_the_Grenadines	13/03/2020	110593	55	0,090909091
San_Marino	31/12/2019	34453	699	0
Sao_Tome_and_Principe	09/04/2020	215048	875	0,147428571
Saudi_Arabia	03/03/2020	34268529	281435	0,09979569
Senegal	03/03/2020	16296362	10432	0,142254601
Serbia	07/03/2020	6963764	26738	0,205138754
Seychelles	15/03/2020	97741	126	0,142857143
Sierra_Leone	01/04/2020	7813207	1855	0,077628032
Singapore	31/12/2019	5804343	53346	0,099557605
Sint_Maarten	03/03/2020	42389	156	0,493589744
Slovakia	07/03/2020	5450421	2368	0,163851351
Slovenia	05/03/2020	2080908	2190	0,108219178
Somalia	17/03/2020	15442906	6447	0,04530389
South_Africa	06/03/2020	58558267	521318	0,283301171
South_Korea	31/12/2019	51225321	14456	0,044272274
South_Sudan	06/04/2020	11062114	2437	0,092736972
Spain	31/12/2019	46937060	302814	0,125416923
Sri_Lanka	31/12/2019	21323734	2834	0,036697248
Sudan	14/03/2020	42813237	11780	0,066893039
Suriname	15/03/2020	581363	1981	0,455325593
Sweden	31/12/2019	10230185	77777	0,041644702
Switzerland	31/12/2019	8544527	35657	0,059174917
Syria	23/03/2020	17070132	892	0,414798206
Taiwan	31/12/2019	23773881	476	0,044117647
Tajikistan	01/05/2020	9321023	7583	0,087300541
Thailand	31/12/2019	69625581	3328	0,021935096
Timor_Leste	22/03/2020	1293120	25	0,04
Togo	07/03/2020	8082359	988	0,207489879
Trinidad_and_Tobago	13/03/2020	1394969	194	0,293814433
Tunisia	03/03/2020	11694721	1584	0,128156566
Turkey	12/03/2020	82003882	234934	0,061132063
Turks_and_Caicos_islands	25/03/2020	38194	116	0,301724138
Uganda	22/03/2020	44269587	1203	0,111388196
Ukraine	04/03/2020	43993643	74219	0,198412805
United_Arab_Emirates	31/12/2019	9770526	61352	0,067789151
United_Kingdom	31/12/2019	66647112	306293	0,03322962
United_Republic_of_Tanzania	17/03/2020	58005461	509	0
United_States_of_America	31/12/2019	329064917	4771087	0,197245827
United_States_Virgin_Islands	24/03/2020	104579	463	0,334773218

	Min. dateRep	Max. popData2019	Cumulative cases at 05-10-2020	% increase incidence last 15 days.
Uruguay	15/03/2020	3461731	1300	0,181538462
Uzbekistan	16/03/2020	32981715	27314	0,356007908
Venezuela	15/03/2020	28515829	21438	0,42466648
Vietnam	31/12/2019	96462108	672	0,444940476
Western_Sahara	26/04/2020	582458	766	0
Yemen	10/04/2020	29161922	1760	0,080113636
Zambia	19/03/2020	17861034	6580	0,494528875
Zimbabwe	21/03/2020	14645473	4221	0,594171997
Solomon_Islands	16/10/2020	669821	3	0
	**31/12/2019**	**1433783692**	**18581702**	**36,64331222**

S1 COVID-19 - Geographic-disbtribution-worldwide _ incidence speed population exposure risk

	country	geo Id	date 1° report	cases	Speed	c/100000p	population	Log(10)Incidence	Exposure Risk	Potential Damage	
1	India	IN	31/12/2019	1.908.254	39,5%	139,7	1.366.417.756	2,145053	0,308061	0,2936	
2	United_States_	US	31/12/2019	4.771.087	19,7%	1.449,9	329.064.917	3,161336	0,211941	0,0486	
3	Brazil	BR	31/12/2019	2.801.921	24,4%	1.327,6	211.049.519	3,123072	0,25933	0,0382	
4	Philippines	PH	31/12/2019	112.593	38,8%	104,1	108.116.622	2,017619	0,288643	0,0218	
5	Japan	JP	31/12/2019	41.129	37,4%	32,4	126.860.299	1,510822	0,223517	0,0198	
6	Colombia	CO	07/03/2020	334.979	39,1%	665,4	50.339.443	2,823109	0,381881	0,0134	
7	South_Africa	ZA	06/03/2020	521.318	28,3%	890,3	58.558.267	2,949515	0,287055	0,0117	
8	Argentina	AR	04/03/2020	206.730	38,7%	461,7	44.780.675	2,664313	0,360137	0,0112	
9	Iraq	IQ	31/12/2019	134.722	29,7%	342,7	39.309.789	2,534938	0,26544	0,0073	
10	Morocco	MA	03/03/2020	27.217	35,5%	74,6	36.471.766	1,872883	0,249	0,0063	
11	Peru	PE	07/03/2020	439.890	18,7%	1.353,1	32.510.462	3,131321	0,199187	0,0045	
12	Australia	AU	31/12/2019	18.729	35,6%	74,3	25.203.200	1,871059	0,249414	0,0044	
13	Romania	RO	31/12/2019	55.241	31,0%	284,5	19.414.458	2,454136	0,269343	0,0036	
14	Dominican_Rep	DO	31/12/2019	74.295	27,4%	691,8	10.738.957	2,839997	0,268718	0,0020	
15	Israel	IL	31/12/2019	76.642	32,6%	899,6	8.519.373	2,954059	0,330493	0,0020	
16	Lebanon	LB	31/12/2019	5.062	42,6%	73,8	6.855.709	1,86827	0,298531	0,0014	
17	Costa_Rica	CR	07/03/2020	19.837	41,9%	393,0	5.047.561	2,594394	0,381125	0,0013	
18	Paraguay	PY	08/03/2020	5.852	36,0%	83,1	7.044.639	1,919446	0,257206	0,0013	
19	Palestine	PS	06/03/2020	16.628	36,7%	333,8	4.981.422	2,523487	0,326946	0,0011	
20	Bulgaria	BG	08/03/2020	12.159	26,6%	173,7	7.000.039	2,239797	0,214648	0,0010	
21	Panama	PA	10/03/2020	69.424	21,6%	1.634,9	4.246.440	3,213485	0,235388	0,0007	
22	Bosnia_and_He	BA	06/03/2020	12.858	33,2%	389,5	3.300.998	2,590528	0,301804	0,0007	
23	Albania	AL	09/03/2020	5.750	27,5%	200,9	2.862.427	2,302933	0,226903	0,0005	
24	Maldives	MV	08/03/2020	4.446	33,3%	837,4	530.957	2,92291	0,334734	0,0001	
25	Luxembourg	LU	31/12/2019	6.917	19,0%	1.126,7	613.894	3,051824	0,197803	0,0001	
26	Sint_Maarten	SX	03/03/2020	156	49,4%	368,0	42.389	2,565871	0,445372	0,0000	
27	Indonesia	ID	31/12/2019	115.056	23,3%	42,5	270.625.567	1,62854	0,14727	0,0278	1
28	Mexico	MX	31/12/2019	502.966	19,7%	394,2	127.575.529	2,595771	0,179552	0,0160	2
29	Bangladesh	BD	09/03/2020	244.020	15,0%	149,7	163.046.173	2,175115	0,118281	0,0135	3
30	Nigeria	NG	31/12/2019	44.433	16,2%	22,1	200.963.603	1,344588	0,089084	0,0125	4
31	Russia	RU	31/12/2019	861.423	9,7%	590,5	145.872.260	2,771244	0,093708	0,0095	5
32	Iran	IR	31/12/2019	314.786	12,3%	379,7	82.913.893	2,579388	0,111077	0,0064	6
33	Pakistan	PK	31/12/2019	281.136	5,3%	129,8	216.565.317	2,113328	0,04127	0,0062	7
34	Algeria	DZ	31/12/2019	32.504	27,1%	75,5	43.053.054	1,877933	0,190711	0,0057	8
35	Ukraine	UA	04/03/2020	74.219	19,8%	168,7	43.993.643	2,227125	0,159595	0,0049	9
36	Spain	ES	31/12/2019	302.814	12,5%	645,1	46.937.060	2,80966	0,122007	0,0040	10
37	Vietnam	VN	31/12/2019	672	44,5%	0,7	96.462.108	-0,156987	0,05114	0,0034	11
38	Egypt	EG	31/12/2019	94.752	6,7%	94,4	100.388.076	1,974906	0,049018	0,0034	12
39	France	FR	31/12/2019	192.334	8,1%	287,0	67.012.883	2,457898	0,070563	0,0033	13
40	Poland	PL	04/03/2020	48.149	16,1%	126,8	37.972.812	2,103115	0,123951	0,0033	14
41	Saudi_Arabia	SA	03/03/2020	281.435	10,0%	821,3	34.268.529	2,914483	0,100107	0,0024	15
42	Nepal	NP	31/12/2019	21.009	15,1%	73,4	28.608.715	1,865907	0,10544	0,0021	16
43	Ecuador	EC	31/12/2019	87.963	15,2%	506,3	17.373.657	2,704409	0,142947	0,0017	17
44	Canada	CA	31/12/2019	117.777	5,7%	314,8	37.411.038	2,498061	0,049945	0,0013	18
45	Chile	CL	04/03/2020	362.962	8,2%	1.915,2	18.952.035	3,282205	0,091495	0,0012	19
46	Senegal	SN	03/03/2020	10.432	14,2%	64,0	16.296.362	1,806277	0,097111	0,0011	20
47	Cameroon	CM	07/03/2020	17.718	8,8%	68,5	25.876.387	1,835511	0,060889	0,0011	21
48	Czechia	CZ	31/12/2019	17.286	18,4%	162,3	10.649.800	2,210353	0,14745	0,0011	22
49	Azerbaijan	AZ	31/12/2019	32.910	15,3%	327,5	10.047.719	2,51526	0,135404	0,0009	23

	country	geo Id	date 1° report	cases	Speed	c/100000 p	population	Log(10)Incidence	Exposure Risk	Potential Damage	
50	Serbia	RS	07/03/2020	26.738	20,5%	384,0	6.963.764	2,584285	0,186192	0,0009	24
51	Greece	EL	31/12/2019	4.855	17,4%	45,3	10.724.599	1,655808	0,110979	0,0008	25
52	Belgium	BE	31/12/2019	72.338	10,2%	631,5	11.455.519	2,800352	0,099402	0,0008	26
53	Netherlands	NL	31/12/2019	55.955	7,2%	323,8	17.282.163	2,510241	0,064065	0,0008	27
54	Togo	TG	07/03/2020	988	20,7%	12,2	8.082.359	1,087219	0,098501	0,0006	28
55	Oman	OM	31/12/2019	79.159	13,6%	1.591,1	4.974.992	3,201708	0,147629	0,0005	29
56	Tunisia	TN	03/03/2020	1.584	12,8%	13,5	11.694.721	1,131765	0,06249	0,0005	30
57	Moldova	MD	08/03/2020	25.814	18,2%	638,4	4.043.258	2,805124	0,176845	0,0005	31
58	United_Arab_E	AE	31/12/2019	61.352	6,8%	627,9	9.770.526	2,797911	0,065716	0,0004	32
59	Austria	AT	31/12/2019	21.472	8,4%	242,4	8.858.775	2,384499	0,070967	0,0004	33
60	Kuwait	KW	31/12/2019	68.774	13,1%	1.634,7	4.207.077	3,213444	0,142759	0,0004	34
61	Singapore	SG	31/12/2019	53.346	10,0%	919,1	5.804.343	2,963349	0,101275	0,0004	35
62	Slovakia	SK	07/03/2020	2.368	16,4%	43,4	5.450.421	1,637952	0,103879	0,0004	36
63	Croatia	HR	31/12/2019	5.318	17,8%	130,5	4.076.246	2,115488	0,137632	0,0004	37
64	Portugal	PT	03/03/2020	51.681	5,6%	502,9	10.276.617	2,701481	0,053015	0,0004	38
65	Switzerland	CH	31/12/2019	35.657	5,9%	417,3	8.544.527	2,620457	0,054328	0,0003	39
66	Sweden	SE	31/12/2019	77.777	4,2%	760,3	10.230.185	2,880968	0,041371	0,0003	40
67	North_Macedo	MK	31/12/2019	11.140	17,0%	536,3	2.077.132	2,729421	0,161194	0,0002	41
68	Armenia	AM	31/12/2019	39.298	10,3%	1.328,7	2.957.728	3,123412	0,109444	0,0002	42
69	Georgia	GE	31/12/2019	1.197	13,2%	29,9	3.996.762	1,476386	0,077517	0,0002	43
70	Denmark	DK	31/12/2019	14.073	5,8%	242,4	5.806.081	2,384504	0,048976	0,0002	44
71	Bahrain	BH	31/12/2019	42.132	12,3%	2.567,2	1.641.164	3,40946	0,141364	0,0002	45
72	Lithuania	LT	31/12/2019	2.137	8,9%	76,5	2.794.184	1,88355	0,062682	0,0001	46
73	Slovenia	SI	05/03/2020	2.190	10,8%	105,2	2.080.908	2,022191	0,080634	0,0001	47
74	Qatar	QA	31/12/2019	111.538	4,0%	3.938,4	2.832.071	3,595319	0,048425	0,0001	48
75	Cyprus	CY	10/03/2020	1.180	12,0%	134,7	875.899	2,129428	0,093396	0,0001	49
76	Malta	MT	07/03/2020	799	15,5%	161,9	493.559	2,209208	0,124027	0,0000	50
77	Bhutan	BT	06/03/2020	105	14,3%	13,8	763.094	1,138611	0,069941	0,0000	51
78	Faroe_Islands	FO	06/03/2020	227	15,9%	466,3	48.677	2,668702	0,147814	0,0000	52
79	Andorra	AD	03/03/2020	939	5,9%	1.232,7	76.177	3,090842	0,061743	0,0000	53
80	Monaco	MC	31/12/2019	87	12,6%	263,0	33.085	2,419888	0,108748	0,0000	54
81	China	CN	31/12/2019	88.206	2,8%	6,2	1.433.783.692	0,789014	0,011051	0,0111	1
82	Germany	DE	31/12/2019	212.022	4,6%	255,4	83.019.213	2,407202	0,039089	0,0023	2
83	United_Kingdo	UK	31/12/2019	306.293	3,3%	459,6	66.647.112	2,662356	0,030911	0,0014	3
84	South_Korea	KR	31/12/2019	14.456	4,4%	28,2	51.225.321	1,450563	0,025669	0,0009	4
85	Afghanistan	AF	31/12/2019	36.829	3,3%	96,8	38.041.757	1,985929	0,024215	0,0006	5
86	Italy	IT	31/12/2019	248.419	1,5%	411,6	60.359.546	2,614439	0,013999	0,0006	6
87	Cambodia	KH	31/12/2019	243	18,9%	1,5	16.486.542	0,168477	0,039574	0,0005	7
88	Thailand	TH	31/12/2019	3.328	2,2%	4,8	69.625.581	0,679414	0,007826	0,0004	8
89	Malaysia	MY	31/12/2019	9.002	2,2%	28,2	31.949.789	1,449871	0,013006	0,0003	9
90	Sri_Lanka	LK	31/12/2019	2.834	3,7%	13,3	21.323.734	1,123537	0,017807	0,0003	10
91	Hungary	HU	05/03/2020	4.553	4,5%	46,6	9.772.756	1,668281	0,029081	0,0002	11
92	Belarus	BY	31/12/2019	68.250	3,0%	722,0	9.452.409	2,85856	0,029457	0,0002	12
93	Taiwan	TW	31/12/2019	476	4,4%	2,0	23.773.881	0,301507	0,01092	0,0002	13
94	Norway	NO	31/12/2019	9.333	3,3%	175,2	5.328.212	2,24344	0,02644	0,0001	14
95	Ireland	IE	31/12/2019	26.253	1,9%	535,3	4.904.240	2,728607	0,017611	0,0001	15
96	Finland	FI	31/12/2019	7.483	1,9%	135,6	5.517.919	2,1323	0,014847	0,0001	16
97	Latvia	LV	03/03/2020	1.249	4,6%	65,1	1.919.968	1,813268	0,031246	0,0000	17
98	Estonia	EE	31/12/2019	2.091	3,3%	157,8	1.324.820	2,198197	0,026647	0,0000	18
99	New_Zealand	NZ	31/12/2019	1.219	1,1%	25,5	4.783.062	1,406298	0,006512	0,0000	19
100	Mongolia	MN	10/03/2020	293	2,0%	9,1	3.225.166	0,958316	0,008958	0,0000	20

	country	geo Id	date 1° report	cases	Speed	c/100000 p	population	Log(10)Incidence	Exposure Risk	Potential Damage	
101	Iceland	IS	31/12/2019	1.918	4,1%	537,3	356.991	2,730191	0,039122	0,0000	21
102	Jordan	JO	03/03/2020	1.224	0,1%	12,1	10.101.697	1,083387	0,000387	0,0000	22
103	Liechtenstein	LI	05/03/2020	89	2,2%	231,9	38.378	2,365308	0,018973	0,0000	23
104	San_Marino	SM	31/12/2019	699	0,0%	2.028,9	34.453	3,30725	0	-	24
105	Brunei_Darussa	BN	10/03/2020	141	0,0%	32,5	433.296	1,512434	0	-	25
106	Holy_See	VA	07/03/2020	12	0,0%	1.472,4	815	3,168024	0	-	26
107	Democratic_Re	CD	11/03/2020	9.177	8,0%	10,6	86.790.568	1,024228	0,036563	0,0022	
108	Burkina_Faso	BF	11/03/2020	1.156	7,9%	5,7	20.321.383	0,755005	0,029808	0,0004	
109	Turkey	TR	12/03/2020	234.934	6,1%	286,5	82.003.882	2,457111	0,053238	0,0030	
110	Bolivia	BO	12/03/2020	83.361	26,8%	724,1	11.513.102	2,859771	0,264943	0,0021	
111	Honduras	HN	12/03/2020	44.299	21,9%	454,5	9.746.115	2,657562	0,20313	0,0014	
112	Cuba	CU	12/03/2020	2.701	9,4%	23,8	11.333.484	1,377161	0,052734	0,0004	
113	Cote_dIvoire	CI	12/03/2020	16.293	12,2%	63,4	25.716.554	1,801788	0,082844	0,0015	
114	Jamaica	JM	12/03/2020	920	12,1%	31,2	2.948.277	1,49422	0,071477	0,0001	
115	Ghana	GH	13/03/2020	37.812	24,8%	124,3	30.417.858	2,094501	0,190063	0,0040	
116	Trinidad_and_T	TT	13/03/2020	194	29,4%	13,9	1.394.969	1,143237	0,144241	0,0001	
117	Guyana	GY	13/03/2020	497	32,2%	63,5	782.775	1,802719	0,219438	0,0001	
118	Gabon	GA	13/03/2020	7.646	15,9%	351,9	2.172.578	2,546459	0,142257	0,0002	
119	Aruba	AW	13/03/2020	132	12,9%	124,2	106.310	2,094	0,098634	0,0000	
120	CuraÃ§ao	CW	13/03/2020	29	3,4%	17,7	163.423	1,249085	0,017984	0,0000	
121	Saint_Vincent_	VC	13/03/2020	55	9,1%	49,7	110.593	1,696635	0,059178	0,0000	
122	Ethiopia	ET	14/03/2020	19.877	48,6%	17,7	112.078.727	1,248828	0,253686	0,0198	
123	Kenya	KE	14/03/2020	23.202	40,6%	44,1	52.573.967	1,644755	0,258497	0,0095	
124	Sudan	SD	14/03/2020	11.780	6,7%	27,5	42.813.237	1,439567	0,038572	0,0012	
125	Guinea	GN	14/03/2020	7.489	12,0%	58,6	12.771.246	1,768191	0,080626	0,0007	
126	Kosovo	XK	14/03/2020	9.274	36,6%	515,7	1.798.506	2,712355	0,346026	0,0004	
127	Venezuela	VE	15/03/2020	21.438	42,5%	75,2	28.515.829	1,876098	0,298476	0,0059	
128	Kazakhstan	KZ	15/03/2020	102.936	28,6%	554,9	18.551.428	2,74419	0,273071	0,0035	
129	Guatemala	GT	15/03/2020	52.365	25,4%	297,8	17.581.476	2,473986	0,222861	0,0027	
130	Rwanda	RW	15/03/2020	2.099	22,4%	16,6	12.626.938	1,220714	0,114943	0,0010	
131	Namibia	NA	15/03/2020	2.470	45,6%	99,0	2.494.524	1,995709	0,336176	0,0006	
132	Equatorial_Gui	GQ	15/03/2020	4.821	36,3%	355,5	1.355.982	2,550883	0,325962	0,0003	
133	Uruguay	UY	15/03/2020	1.300	18,2%	37,6	3.461.731	1,57465	0,111769	0,0003	
134	Eswatini	SZ	15/03/2020	2.856	36,1%	248,8	1.148.133	2,395766	0,307674	0,0002	
135	Mauritania	MR	15/03/2020	6.382	7,2%	141,0	4.525.698	2,149271	0,056231	0,0002	
136	Suriname	SR	15/03/2020	1.981	45,5%	340,8	581.363	2,532437	0,406444	0,0002	
137	Seychelles	SC	15/03/2020	126	14,3%	128,9	97.741	2,110294	0,110082	0,0000	
138	Saint_Lucia	LC	15/03/2020	25	8,0%	13,7	182.795	1,135976	0,039106	0,0000	
139	Antigua_and_B	AG	15/03/2020	92	17,4%	94,7	97.115	1,976502	0,127284	0,0000	
140	Uzbekistan	UZ	16/03/2020	27.314	35,6%	82,8	32.981.715	1,918112	0,254545	0,0059	
141	Congo	CG	16/03/2020	3.546	19,6%	65,9	5.380.504	1,818916	0,134514	0,0005	
142	Bahamas	BS	16/03/2020	715	75,7%	183,6	389.486	2,263814	0,616638	0,0002	
143	Central_African	CF	16/03/2020	4.618	1,5%	97,3	4.745.179	1,988201	0,011145	0,0000	
144	Myanmar	MM	17/03/2020	356	4,2%	0,7	54.045.422	-0,181309	0,004547	0,0002	
145	Benin	BJ	17/03/2020	1.914	16,3%	16,2	11.801.151	1,210018	0,083173	0,0007	
146	Somalia	SO	17/03/2020	6.447	4,5%	41,7	15.442.906	1,620629	0,028495	0,0003	
147	Liberia	LR	17/03/2020	1.216	9,0%	24,6	4.937.374	1,391438	0,050439	0,0002	
148	United_Republi	TZ	17/03/2020	509	0,0%	0,9	58.005.461	-0,056751	0	-	
149	Gambia	GM	18/03/2020	671	83,3%	28,6	2.347.696	1,456081	0,484349	0,0008	
150	Montenegro	ME	18/03/2020	3.361	44,6%	540,2	622.182	2,732551	0,424209	0,0002	
151	Barbados	BB	18/03/2020	132	19,7%	46,0	287.021	1,66266	0,126283	0,0000	

	country	geo Id	date 1° report	cases	Speed	c/100000 p	population	Log(10)Incidence	Exposure Risk	Potential Damage
152	Zambia	ZM	19/03/2020	6.580	49,5%	36,8	17.861.034	1,566319	0,30328	0,0038
153	El_Salvador	SV	19/03/2020	18.262	33,2%	283,0	6.453.550	2,45175	0,288231	0,0013
154	Kyrgyzstan	KG	19/03/2020	38.110	25,9%	594,0	6.415.851	2,773785	0,248981	0,0011
155	Nicaragua	NI	19/03/2020	3.902	19,3%	59,6	6.545.503	1,775344	0,130357	0,0006
156	French_Polynes	PF	19/03/2020	62	0,0%	22,2	279.285	1,346344	0	-
157	Guam	GU	19/03/2020	389	15,9%	232,5	167.295	2,366467	0,134623	0,0000
158	Djibouti	DJ	19/03/2020	5.248	4,3%	539,1	973.557	2,731632	0,041284	0,0000
159	Haiti	HT	20/03/2020	7.532	6,2%	66,9	11.263.079	1,825253	0,042849	0,0003
160	Chad	TD	20/03/2020	938	5,2%	5,9	15.946.882	0,769527	0,02	0,0002
161	Gibraltar	GI	20/03/2020	189	4,8%	560,7	33.706	2,748755	0,045486	0,0000
162	Jersey	JE	20/03/2020	343	3,5%	318,2	107.796	2,502691	0,030929	0,0000
163	Fiji	FJ	20/03/2020	27	3,7%	3,0	889.955	0,481996	0,0111	0,0000
164	Mauritius	MU	20/03/2020	344	0,3%	27,1	1.269.670	1,432868	0,001671	0,0000
165	Bermuda	BM	20/03/2020	157	2,5%	251,2	62.508	2,399964	0,021767	0,0000
166	Cayman_Island	KY	20/03/2020	416	1,4%	640,5	64.948	2,806528	0,013689	0,0000
167	Guernsey	GG	20/03/2020	252	0,0%	390,9	64.468	2,592056	0	-
168	Greenland	GL	20/03/2020	14	7,1%	24,7	56.660	1,392851	0,040222	0,0000
169	Madagascar	MG	21/03/2020	11.895	39,9%	44,1	26.969.306	1,644495	0,253495	0,0048
170	Zimbabwe	ZW	21/03/2020	4.221	59,4%	28,8	14.645.473	1,459712	0,346071	0,0035
171	Papua_New_Gu	PG	21/03/2020	114	83,3%	1,3	8.776.119	0,113602	0,160987	0,0010
172	Niger	NE	21/03/2020	1.152	4,1%	4,9	23.310.719	0,693897	0,014728	0,0002
173	Cape_Verde	CV	21/03/2020	2.631	21,3%	478,4	549.936	2,679809	0,199067	0,0001
174	New_Caledonia	NC	21/03/2020	22	0,0%	7,8	282.757	0,891009	0	-
175	Isle_of_Man	IM	21/03/2020	336	0,0%	397,2	84.589	2,599025	0	-
176	Montserrat	MS	21/03/2020	13	7,7%	260,5	4.991	2,415756	0,066069	0,0000
177	Uganda	UG	22/03/2020	1.203	11,1%	2,7	44.269.587	0,43416	0,031844	0,0010
178	Angola	AO	22/03/2020	1.164	35,7%	3,7	31.825.299	0,56318	0,115227	0,0026
179	Eritrea	ER	22/03/2020	282	11,0%	8,1	3.497.117	0,906539	0,046443	0,0001
180	Timor_Leste	TL	22/03/2020	25	4,0%	1,9	1.293.120	0,286301	0,009725	0,0000
181	Mozambique	MZ	23/03/2020	2.029	25,7%	6,7	30.366.043	0,824894	0,102617	0,0022
182	Syria	SY	23/03/2020	892	41,5%	5,2	17.070.132	0,718128	0,152644	0,0018
183	Dominica	DM	23/03/2020	18	0,0%	25,1	71.808	1,3991	0	-
184	Grenada	GD	23/03/2020	24	4,2%	21,4	112.002	1,330985	0,022717	0,0000
185	Belize	BZ	24/03/2020	72	41,7%	18,4	390.351	1,265877	0,219329	0,0001
186	United_States_	VI	24/03/2020	463	33,5%	442,7	104.579	2,646137	0,30984	0,0000
187	Libya	LY	25/03/2020	4.063	51,8%	59,9	6.777.453	1,77778	0,349077	0,0017
188	Turks_and_Caic	TC	25/03/2020	116	30,2%	303,7	38.194	2,482463	0,264972	0,0000
189	Laos	LA	25/03/2020	20	5,0%	0,3	7.169.456	-0,554456	0	-
190	Mali	ML	26/03/2020	2.543	2,7%	12,9	19.658.023	1,111807	0,012884	0,0002
191	Saint_Kitts_and	KN	26/03/2020	17	0,0%	32,2	52.834	1,507535	0	-
192	Guinea_Bissau	GW	27/03/2020	2.032	4,0%	105,8	1.920.917	2,024415	0,030094	0,0000
193	British_Virgin_I	VG	27/03/2020	9	11,1%	30,0	30.033	1,476644	0,06526	0,0000
194	Anguilla	AI	27/03/2020	3	0,0%	20,2	14.872	1,304752	0	-
195	Puerto_Rico	PR	28/03/2020	19.324	35,5%	658,8	2.933.404	2,818725	0,346428	0,0007
196	Northern_Mari	MP	31/03/2020	46	17,4%	80,4	57.213	1,905263	0,123701	0,0000
197	Botswana	BW	01/04/2020	804	35,1%	34,9	2.303.703	1,54283	0,21272	0,0003
198	Burundi	BI	01/04/2020	395	18,5%	3,4	11.530.577	0,534746	0,058209	0,0005
199	Sierra_Leone	SL	01/04/2020	1.855	7,8%	23,7	7.813.207	1,375515	0,043324	0,0002
200	Bonaire, Saint E	BQ	02/04/2020	13	23,1%	50,0	25.983	1,699254	0,150395	0,0000
201	Malawi	MW	03/04/2020	4.347	30,0%	23,3	18.628.749	1,368006	0,166508	0,0022
202	Falkland_Island	FK	04/04/2020	13	0,0%	385,5	3.372	2,586056	0	-

	country	geo Id	date 1° report	cases	Speed	c/100000 p	population	Log(10)Incidence	Exposure Risk	Potential Damage
203	South_Sudan	SS	06/04/2020	2.437	9,3%	22,0	11.062.114	1,343017	0,050884	0,0004
204	Sao_Tome_and	ST	09/04/2020	875	14,7%	406,9	215.048	2,609473	0,134885	0,0000
205	Yemen	YE	10/04/2020	1.760	8,0%	6,0	29.161.922	0,780697	0,030931	0,0006
206	Western_Sahar	EH	26/04/2020	766	0,0%	131,5	582.458	2,118964	0	-
207	Tajikistan	TJ	01/05/2020	7.583	8,7%	81,4	9.321.023	1,910377	0,062224	0,0004
208	Comoros	KM	02/05/2020	388	13,9%	45,6	850.891	1,658958	0,08908	0,0001
209	Lesotho	LS	15/05/2020	726	50,6%	34,2	2.125.267	1,533523	0,30522	0,0005
210	Solomon_Islanc	SB	16/10/2020	3	0,0%	0,4	669.821	-0,348838	0	-
	Cases_on_an_i	JPG:	31/12/2019	-	0,0%		-			
