# Supplementary material for: The exposure risk to COVID-19 in most affected countries: A vulnerability assessment model
Source: PLoS One. 2021 Mar 4;16(3):e0248075. doi: 10.1371/journal.pone.0248075 (PMC7932136; doi:10.1371/journal.pone.0248075)
Supplement: S2 File — (PDF) [file pone.0248075.s002.pdf]

INDICATORS SENSITIVITY

												Indicator variation							Position								Sensitivity
Dataset	Class	Factors		INDICATOR	Year	Brazil	US	India	China	Germany	New Zealand	BR	US	IN	CN	DE	NZ	RIG	BR	US	IN	CN	DE	NZ	RPGI	S	Cluster
IBGE	Socioeconomic	Demographic	1 DM	Population density (IBGE)	2015	24.46	34.97	440.66	149.85	234.64	17.53	0.98	0.96	0.00	0.69	0.49	1.00	3.23	2	3	6	4	5	1	10	10.32	Low
IBGE	Socioeconomic	Demographic	2 DM	Population living in urban areas (IBGE)	2019	86.8	82.5	34.5	60.3	77.4	86.6	0.00	0.08	1.00	0.51	0.18	0.00	2.61	6	4	1	2	3	5	10	10.26	Low
IBGE	Socioeconomic	Demographic	3 DM	Average annual population growth rate (IBGE)	2015-2020	0.753	0.706	1.102	0.39	0.203	0.931	0.39	0.44	0.00	0.79	1.00	0.19	4.15	4	3	6	2	1	5	14	14.42	Medium
IBGE	Socioeconomic	Demographic	4 DM	Literacy rate - population aged 15 and over (IBGE)	2018	93.2275	..	74.373	96.8409	..	..	0.84		0.00	1.00			3.16	2		3	1			13	13.32	Medium
IBGE	Socioeconomic	Demographic	5 DM	Gross enrollment rate all school levels (IBGE)	2017	90.84739	98.3794	72.08312	78.79346	96.22452	104.03514	0.59	0.82	0.00	0.21	0.76	1.00	3.56	4	2	6	5	3	1	12	12.36	Medium
WB	Socioeconomic	Demographic	6 DM	Population growth (annual % (World Bank))	2018	0.8	0.5	1	0.5	0.3	1	0.71	0.29	1.00	0.29	0.00	1.00	2.29	3	4	1	4	6	1	6	6.23	No
WB	Socioeconomic	Demographic	7 DM	Net migration (thousands (World Bank))	2018	106	4774	-2663	-1742	2719	74	0.37	1.00	0.00	0.12	0.72	0.37	2.84	3	1	6	5	2	4	8	8.28	Low
WHO	Socioeconomic	Demographic	8 DM	Population median age (years (WHO))	2013	30.3	37.4	26.4	37.4	45.5	37	0.20	0.58	0.00	0.58	1.00	0.55	4.35	5	2	6	2	1	4	15	15.44	High
WB	Socioeconomic	Demographic	9 DM	Income share held by lowest 20% (World Bank)	2018	3.1	5.1	..	6.5	7.6	..	0.00	0.44		0.76	1.00		4.31	4	3		2	1		13	13.43	Medium
WB	Socioeconomic	Demographic	10 DM	Fertility rate, total (births per woman (World Bank))	2018	1.7	1.7	2.2	1.7	1.6	1.7	0.83	0.83	0.00	0.83	1.00	0.83	4.00	2	2	6	2	1	2	14	14.40	Medium
WB	Socioeconomic	Demographic	11 DM	Adolescent fertility rate (births per 1000 women ages 15-19 (World Bank))	2018	58	19	12	8	8	19	0.00	0.78	0.92	1.00	1.00	0.78	4.08	6	4	3	1	1	4	16	16.41	High
IBGE	Socioeconomic	Demographic	12 DM	Life expectancy at birth (IBGE)	2018	75.7	78.9	69.4	76.7	81.2	82.1	0.50	0.75	0.00	0.57	0.93	1.00	4.26	5	3	6	4	2	1	16	16.43	High
IBGE	Socioeconomic	Demographic	13 DM	Human development Index (IBGE)	2018	0.761	0.92	0.647	0.758	0.939	0.921	0.39	0.93	0.00	0.38	1.00	0.94	3.99	4	3	6	5	1	2	14	14.40	Medium
IBGE	Socioeconomic	Economic	14 EC	GDP per capita (IBGE)	2015	8814	57938	1726	7830	41089	38458	0.13	1.00	0.00	0.11	0.70	0.65	3.34	4	1	6	5	2	3	10	10.33	Low
WB	Socioeconomic	Economic	15 EC	GDP per capita growth (annual % (World Bank))	2019	0.3793442	1.8497114	3.9631625	5.7301207	0.2895567	0.5917646	0.02	0.29	0.68	1.00	0.00	0.06	3.08	5	3	2	1	6	4	8	8.31	Low
WB	Socioeconomic	Economic	16 EC	GDP growth (annual % (World Bank))	2018	1.3	3.2	6.1	6.8	1.5	3.8	0.00	0.35	0.87	1.00	0.04	0.45	3.27	6	4	2	1	5	3	12	12.33	Medium
WB	Socioeconomic	Economic	17 EC	Inflation, GDP deflator (annual % (World Bank))	2018	3.3	2.4	4.6	3.5	1.5	0.2	0.30	0.50	0.00	0.25	0.70	1.00	4.16	4	3	6	5	2	1	14	14.42	Medium
WB	Socioeconomic	Economic	18 EC	Poverty headcount ratio at $1.90 a day (2011 PPP (% of population (World Ba	2018	4.4	1.2	..	0.5	..	..	0.00	0.82		1.00			3.18	3	2		1			13	13.32	Medium
WB	Socioeconomic	Economic	19 EC	Partner-nation transportation infrastructure: Logistics performance index: Ov	2018	2.99	3.89	3.18	3.61	4.2	3.88	0.00	0.74	0.16	0.51	1.00	0.74	4.35	6	2	5	4	1	3	14	14.43	Medium
Chartsbin	Socioeconomic	Economic	20 EC	Infrastructure and technology: Partner-nation transportation infrastructure: I	2015	6	63	47	43.5	99.1	63	0.00	0.61	0.44	0.40	1.00	0.61	3.96	6	2	4	5	1	2	13	13.40	Medium
WB	Socioeconomic	Economic	21 EC	Agriculture, forestry, and fishing, value added (% of GDP (World Bank))	2018	4	1	15	7	1	6	0.79	1.00	0.00	0.57	1.00	0.64	3.43	3	1	6	5	1	4	9	9.34	Low
WB	Socioeconomic	Economic	22 EC	Industry (including construction), value added (% of GDP (World Bank))	2018	18	18	26	40	27	20	0.00	0.00	0.36	1.00	0.41	0.09	4.14	5	5	3	1	2	4	15	15.41	High
OECD	Socioeconomic	Economic	23 EC	Index of Technological Sophistication (OECD)	2007	2.49	..	2.91	3.75	..	..	0.00		0.33	1.00			3.67	3		2	1			13	13.37	Medium
WB	Socioeconomic	Economic	24 EC	High-technology exports (% of manufactured exports (World Bank))	2018	13	19	9	31	16	10	0.18	0.45	0.00	1.00	0.32	0.05	3.73	4	2	6	1	3	5	12	12.37	Medium
IBGE	Socioeconomic	Economic	25 EC	Investments in research and development (IBGE)	2015	1.343	2.772	0.62	2.056	2.917	1.23	0.31	0.94	0.00	0.63	1.00	0.27	3.64	4	2	6	3	1	5	12	12.36	Medium
WB	Socioeconomic	Economic	26 EC	Mobile cellular subscriptions (per 100 people (World Bank))	2018	98.8	129	86.9	115.5	129.3	134.9	0.25	0.88	0.00	0.60	0.88	1.00	4.35	5	3	6	4	2	1	16	16.44	High
WB	Socioeconomic	Economic	27 EC	Individuals using the Internet (% of population (World Bank))	2018	70.4	87.3	34.5	54.3	89.7	90.8	0.64	0.94	0.00	0.35	0.98	1.00	3.76	4	3	6	5	2	1	14	14.38	Medium
IBGE	Socioeconomic	Economic	28 EC	Tourist arrivals (IBGE)	2016	6547000	76941000	14570000	59270000	35555000	3370000	0.96	0.00	0.85	0.24	0.56	1.00	3.00	2	6	3	5	4	1	10	10.30	Low
IBGE	Socioeconomic	Economic	29 EC	Public spending on education (IBGE)	2012	5.9	4.9	3.8	0	4.9	7.2	0.82	0.68	0.53	0.00	0.68	1.00	2.65	2	3	5	6	3	1	9	9.27	Low
WB	Socioeconomic	Economic	30 EC	Merchandise trade (% of GDP (World Bank))	2018	23	21	31	33	72	40	0.04	0.00	0.20	0.24	1.00	0.37	4.37	5	6	4	3	1	2	18	18.44	High
WB	Socioeconomic	Economic	31 EC	GINI index (World Bank estimate)		53.9	41.4	37.8	38.5	31.9	36.2	0.00	0.57	0.73	0.70	1.00	0.80	4.20	6	5	3	4	1	2	16	16.42	High
WHO	Health Infraestructure	Health Care	32 HC	Skilled health professionals density (per 10 000 population) - latest available year (WHO		93	117	29	60	180	143	0.42	0.58	0.00	0.21	1.00	0.75	3.95	4	3	6	5	1	2	14	14.40	Medium
WB	Health Infraestructure	Health Care	33 HC	Current health expenditure (% of GDP (World Bank))	2017	9.4674766	17.061269	3.5349596	5.1511932	11.246835	9.1700554	0.44	1.00	0.00	0.12	0.57	0.42	2.67	3	1	6	5	2	4	8	8.27	Low
WHO	Health Infraestructure	Health Care	34 HC	Hospital beds (per 10 000 population (WHO))	2012	23	29	4.9	42	83.4	28.3	0.23	0.31	0.00	0.47	1.00	0.30	4.23	5	3	6	2	1	4	16	16.42	High
WB	Health Infraestructure	Health Care	35 HC	Mortality rate, infant (per 1,000 live births (World Bank))	2018	12.8	5.6	29.9	7.4	3.1	4.7	0.64	0.91	0.00	0.84	1.00	0.94	4.24	5	3	6	4	1	2	16	16.42	High
WB	Health Infraestructure	Health Care	36 HC	Mortality rate, under-5 (per 1000 live births (World Bank))	2018	14	7	37	9	4	6	0.70	0.91	0.00	0.85	1.00	0.94	4.18	5	3	6	4	1	2	16	16.42	High
WHO	Health Infraestructure	Health Care	37 HC	Maternal mortality ratio (per 100 000 live births (WHO))	2015	63	18	158	30	5	10	0.62	0.92	0.00	0.84	1.00	0.97	4.27	5	3	6	4	1	2	16	16.43	High
IBGE	Health Infraestructure	Health Care	38 HC	Dietary Energy Supply (IBGE)	2016-2018	3203	3778	2510	3224	3552	3190	0.45	0.00	1.00	0.44	0.18	0.46	2.63	3	6	1	4	5	2	8	8.26	Low
IBGE	Health Infraestructure	Health Care	39 HC	Prevalence of undernourishment (IBGE)	2016-2018	2.5	2.5	14.5	8.5	2.5	2.5	1.00	1.00	0.00	0.50	1.00	1.00	3.50	1	1	6	5	1	1	10	10.35	Low
WB	Health Infraestructure	Public Health	40 PH	Immunization, measles (% of children ages 12-23 months (World Bank))	2018	84	92	90	99	97	92	0.00	0.53	0.40	1.00	0.87	0.53	4.47	6	3	5	1	2	3	17	17.45	High
IBGE	Health Infraestructure	Public Health	41 PH	Population using improved drinking water sources (IBGE)	2017	98.19	99.27	92.67	92.85	100	100	0.75	0.90	0.00	0.02	1.00	1.00	3.37	4	3	6	5	1	1	15	15.34	High
IBGE	Health Infraestructure	Public Health	42 PH	Population using sanitation facilities (IBGE)	2017	88.29	99.97	59.54	84.76	99.23	100	0.71	1.00	0.00	0.62	0.98	1.00	3.89	4	2	6	5	3	1	12	12.39	Medium
IANPHI	Health Infraestructure	Public Health	43 PH	Basic public health infrastructure (Country is member of the International Ass	2019	1	1	1	1	1	0	1.00	1.00	1.00	1.00	1.00	0.00	2.00	1	1	1	1	1	6	4	4.20	No
WHO	Health Infraestructure	Public Health	44 PH	UHC index of essential service coverage (%) - latest available year (scaled (WHO))		76.999998	80.000001	56	75.999999	79.000002	..	0.87	1.00	0.00	0.83	0.96		2.92	3	1	5	4	2		12	12.29	Medium
WHO	Health Infraestructure	Public Health	45 PH	Domestic General Government Health Expenditure (GGHE-D) as % Gross Domestic Prod		0.038	0.085	0.01	0.032	0.094	..	0.33	0.89	0.00	0.26	1.00		3.04	3	2	5	4	1		14	14.30	Medium
IBGE	Health Infraestructure	Public Health	46 PH	Government Health Expenditure (% GDP) (IBGE)	2015	3.806	8.618	0.942	2.942	8.53	6.968	0.37	1.00	0.00	0.26	0.99	0.79	3.66	4	1	6	5	2	3	10	10.37	Low
GHSA	Health Infraestructure	Public Health	47 PH	GHSA action packages (Country leading or contributing to >1 GHSA action pac	2020	0	1	0	1	1	0	0.00	1.00	0.00	1.00	1.00	0.00	4.00	4	1	4	1	1	4	12	12.40	Medium
WB	Health Infraestructure	Disease Dynamics	48 DD	Average precipitation/rainfall in depth (mm per year (World Bank))	2014	1761	715	1083	645	700	1732	0.00	0.94	0.61	1.00	0.95	0.03	3.43	6	3	4	1	2	5	14	14.34	Medium
Weather	Health Infraestructure	Disease Dynamics	49 DD	Annual average temperature (F (Weatherbase))		24.5	11.6	24.2	12.9	8.1	12.2	0.00	0.79	0.02	0.71	1.00	0.75	4.65	6	2	5	4	1	3	14	14.46	Medium
WB	Health Infraestructure	Disease Dynamics	50 DD	Agricultural land (% of land area (World Bank))	2016	33.924533	44.369068	60.447196	56.212313	47.678612	40.45042	0.00	0.39	1.00	0.84	0.52	0.25	3.21	6	4	1	2	3	5	10	10.32	Low
WB	Health Infraestructure	Disease Dynamics	51 DD	Forest area (% of land area (World Bank))	2016	58.931054	33.929786	23.833121	22.353942	32.691207	38.556075	1.00	0.32	0.04	0.00	0.28	0.44	2.37	1	3	5	6	4	2	6	6.24	No
GFW	Health Infraestructure	Disease Dynamics	52 DD	Global deforestation rates (% (Global Forest Watch))	2019	11	14	4.6	6.1	6	11	0.32	0.00	1.00	0.84	0.85	0.32	3.69	4	6	1	3	2	4	11	11.37	Medium
WB	Health Infraestructure	Disease Dynamics	53 DD	Energy use (kg of oil equivalent per capita (World Bank))	2018	..	6806	..	..	3818	4445		1.00			0.00	0.21	2.21		1			3	2	5	5.22	No
WB	Health Infraestructure	Disease Dynamics	54 DD	CO2 emissions (metric tons per capita (World Bank))	2018	2.24	15.5	1.82	7.18	8.84	7.33	0.97	0.00	1.00	0.61	0.49	0.60	2.72	2	6	1	3	5	4	6	6.27	No
WB	Political	Political-Domestic	55 PD	Worldwide Governance Indicator: Government Effectiveness (World Bank)	2018	36.1	92.3	63.9	69.7	93.3	93.8	0.00	0.97	0.48	0.58	0.99	1.00	4.12	6	3	5	4	2	1	16	16.41	High
WB	Political	Political-Domestic	56 PD	Worldwide Governance Indicator: Regulatory Quality (World Bank)	2018	39.9	92.3	46.6	48.1	94.7	98.6	0.00	0.89	0.11	0.14	0.93	1.00	4.07	6	3	5	4	2	1	16	16.41	High
WB	Political	Political-Domestic	57 PD	Worldwide Governance Indicator: Rule of Law Index (World Bank)	2018	44.2	89.4	55.3	48.1	91.3	98.1	0.00	0.84	0.21	0.07	0.87	1.00	3.90	6	3	4	5	2	1	14	14.39	Medium
WB	Political	Political-Domestic	58 PD	Worldwide Governance Indicator: Political Stability and Absence of Violence (	2018	31.9	61.9	14.8	36.7	66.7	99	0.20	0.56	0.00	0.26	0.62	1.00	4.11	5	3	6	4	2	1	16	16.41	High

TI	Political	Political-Domestic	59 PD	Transparency International Corruption Perceptions Index	2019	35	69	41	41	80	87	0.00	0.65	0.12	0.12	0.87	1.00	4.21	6	3	4	4	2	1	15	15.42	High
FSI	Political	Political-Domestic	60 PD	Public Services (Fragile States Index)	2020	6.9	1.2	6.5	4.8	1	0.9	0.00	0.95	0.07	0.35	0.98	1.00	4.32	6	3	5	4	2	1	16	16.43	High
FSI	Political	Political-Domestic	61 PD	State Legitimacy (Fragile States Index)	2020	6.7	2.9	4	8.8	0.5	0.5	0.25	0.71	0.58	0.00	1.00	1.00	3.46	5	3	4	6	1	1	13	13.35	Medium
CSP	Political	Political-Domestic	62 PD	Democracy (Polity IV Project Democracy Index (Center for Systemic Peace))	2018	8	8	9	0	10	10	0.80	0.80	0.90	0.00	1.00	1.00	2.50	4	4	3	6	1	1	12	12.25	Medium
CSP	Political	Political-Domestic	63 PD	Government stability (Fragility Index (Center for Systemic Peace))	2018	6	4	11	7	1	2	0.50	0.70	0.00	0.40	1.00	0.90	4.10	4	3	6	5	1	2	14	14.41	Medium
PTS	Political	Political-Domestic	64 PD	Human rights (Amnesty International Political Terror Scale)	2018	4	3	3	4	1	1	0.00	0.33	0.33	0.00	1.00	1.00	4.33	5	3	3	5	1	1	13	13.43	Medium
WB	Political	Political-International	65 PI	World Bank Net Official Development Assistance per capita	2018	2.0487963	..	1.8142456	-0.529205	..	..	0.00		0.09	1.00			3.91	3		2	1			13	13.39	Medium
WB	Political	Political-International	66 PI	World Bank Net Official Development Assistance received (% gross national ir	2018	0.0234236	..	0.0914221	-0.005324	..	..	0.70		0.00	1.00			3.30	2		3	1			13	13.33	Medium
WB	Political	Political-International	67 PI	World Bank Net financial flows, multilateral (current US$)	2018	737808000	..	1.747E+09	379386000	..	..	0.74		0.00	1.00			3.26	2		3	1			13	13.33	Medium
RAND			IDVI			0.72	0.92	0.49	0.66	0.97	0.92	0.47	0.91	0.00	0.36	1.00	0.89	3.87	4	2	6	5	1	3	12	12.39	Medium
RAND			DM	DG-Demographic Domain Score		0.87266957	0.79641988	0.52727273	0.80322222	0.89021944	0.95494012	0.81	0.63	0.00	0.65	0.85	1.00	4.06	3	5	6	4	2	1	16	16.41	High
RAND			HC	HC-Health Care Domain Score		0.67912958	0.80066552	0.41168754	0.66064964	0.86854247	0.79575444	0.59	0.85	0.00	0.54	1.00	0.84	3.95	4	2	6	5	1	3	12	12.39	Medium
RAND			PH	PH-Public Health Domain Score		0.89172542	0.97031867	0.61104338	0.91245814	0.99509431	0.81892915	0.73	0.94	0.00	0.78	1.00	0.54	3.66	4	2	6	3	1	5	12	12.37	Medium
RAND			DD	DD-Disease Dynamics Domain Score		0.35411132	0.37764899	0.44295234	0.72714874	0.60005594	0.80819022	0.00	0.05	0.20	0.82	0.54	1.00	5.12	6	5	4	2	3	1	18	18.51	High
RAND			PD	PD-Political-Domestic Domain Score		0.54922225	0.89528731	0.47193311	0.34727523	0.91154258	0.96285157	0.33	0.89	0.20	0.00	0.92	1.00	3.50	4	3	5	6	2	1	12	12.35	Medium
RAND			PI	PI-Political- International Domain Score		0.75447225	0.91653193	0.5495857	0.77127555	0.91653193	0.91653193	0.56	1.00	0.00	0.60	1.00	1.00	4.05	5	1	6	4	1	1	15	15.40	High
RAND			EC	EC-Economic Domain Score		0.49092125	0.87614765	0.34224751	0.5247882	0.8631846	0.80260938	0.28	1.00	0.00	0.34	0.98	0.86	3.90	5	1	6	4	2	3	12	12.39	Medium
